# Genetic Architecture of Vitamin B_12_ and Folate Levels Uncovered Applying Deeply Sequenced Large Datasets

**DOI:** 10.1371/journal.pgen.1003530

**Published:** 2013-06-06

**Authors:** Niels Grarup, Patrick Sulem, Camilla H. Sandholt, Gudmar Thorleifsson, Tarunveer S. Ahluwalia, Valgerdur Steinthorsdottir, Helgi Bjarnason, Daniel F. Gudbjartsson, Olafur T. Magnusson, Thomas Sparsø, Anders Albrechtsen, Augustine Kong, Gisli Masson, Geng Tian, Hongzhi Cao, Chao Nie, Karsten Kristiansen, Lise Lotte Husemoen, Betina Thuesen, Yingrui Li, Rasmus Nielsen, Allan Linneberg, Isleifur Olafsson, Gudmundur I. Eyjolfsson, Torben Jørgensen, Jun Wang, Torben Hansen, Unnur Thorsteinsdottir, Kari Stefánsson, Oluf Pedersen

**Affiliations:** 1 The Novo Nordisk Foundation Center for Basic Metabolic Research, Faculty of Health and Medical Sciences, University of Copenhagen, Copenhagen, Denmark; 2 deCODE Genetics, Reykjavik, Iceland; 3 Centre of Bioinformatics, Faculty of Science, University of Copenhagen, Copenhagen, Denmark; 4 BGI-Shenzhen, Shenzhen, China; 5 Department of Biology, Faculty of Science, University of Copenhagen, Copenhagen, Denmark; 6 Research Centre for Prevention and Health, Glostrup University Hospital, Glostrup, Denmark; 7 Department of Integrative Biology, University of California, Berkeley, Berkeley, California, United States of America; 8 Department of Statistics, University of California, Berkeley, Berkeley, California, United States of America; 9 Landspitali, The National University Hospital of Iceland, Department of Clinical Biochemistry, Reykjavik, Iceland; 10 Icelandic Medical Center (Laeknasetrid) Laboratory in Mjodd (RAM), Reykjavik, Iceland; 11 Faculty of Health and Medical Sciences, University of Copenhagen, Copenhagen, Denmark; 12 Faculty of Medicine, University of Aalborg, Aalborg, Denmark; 13 Faculty of Health Sciences, University of Southern Denmark, Odense, Denmark; 14 University of Iceland Faculty of Medicine, Reykjavik, Iceland; 15 Faculty of Health Sciences, Aarhus University, Aarhus, Denmark; 16 Hagedorn Research Institute, Gentofte, Denmark; 17 Institute of Biomedical Science, Faculty of Health and Medical Sciences, University of Copenhagen, Copenhagen, Denmark; Dartmouth College, United States of America

## Abstract

Genome-wide association studies have mainly relied on common HapMap sequence variations. Recently, sequencing approaches have allowed analysis of low frequency and rare variants in conjunction with common variants, thereby improving the search for functional variants and thus the understanding of the underlying biology of human traits and diseases. Here, we used a large Icelandic whole genome sequence dataset combined with Danish exome sequence data to gain insight into the genetic architecture of serum levels of vitamin B_12_ (B_12_) and folate. Up to 22.9 million sequence variants were analyzed in combined samples of 45,576 and 37,341 individuals with serum B_12_ and folate measurements, respectively. We found six novel loci associating with serum B_12_ (*CD320*, *TCN2*, *ABCD4*, *MMAA*, *MMACHC*) or folate levels (*FOLR3*) and confirmed seven loci for these traits (*TCN1*, *FUT6*, *FUT2*, *CUBN*, *CLYBL*, *MUT*, *MTHFR*). Conditional analyses established that four loci contain additional independent signals. Interestingly, 13 of the 18 identified variants were coding and 11 of the 13 target genes have known functions related to B_12_ and folate pathways. Contrary to epidemiological studies we did not find consistent association of the variants with cardiovascular diseases, cancers or Alzheimer's disease although some variants demonstrated pleiotropic effects. Although to some degree impeded by low statistical power for some of these conditions, these data suggest that sequence variants that contribute to the population diversity in serum B_12_ or folate levels do not modify the risk of developing these conditions. Yet, the study demonstrates the value of combining whole genome and exome sequencing approaches to ascertain the genetic and molecular architectures underlying quantitative trait associations.

## Author Summary

Genome-wide association studies have in recent years revealed a wealth of common variants associated with common diseases and phenotypes. We took advantage of the advances in sequencing technologies to study the association of low frequency and rare variants in conjunction with common variants with serum levels of vitamin B_12_ (B_12_) and folate in Icelanders and Danes. We found 18 independent signals in 13 loci associated with serum B_12_ or folate levels. Interestingly, 13 of the 18 identified variants are coding and 11 of the 13 target genes have known functions related to B_12_ and folate pathways. These data indicate that the target genes at all of the loci have been identified. Epidemiological studies have shown a relationship between serum B_12_ and folate levels and the risk of cardiovascular diseases, cancers, and Alzheimer's disease. We investigated association between the identified variants and these diseases but did not find consistent association.

## Introduction

One-carbon metabolism (OCM) is a process whereby folate transfers one-carbon groups in a range of biological processes including DNA synthesis, methylation and homocysteine metabolism [1], [2]. The water-soluble B vitamins, vitamin B_12_ (B_12_) and folate play key roles as enzyme cofactors or substrates in OCM. Individuals with deficiencies in these vitamins can develop anemia and, in the case of B_12_ deficiency, serious neurological problems. In adults, epidemiological studies have also suggested that subclinical B_12_ or folate deficiencies are associated with increased risk of cardiovascular disease [3], [4], different cancers [5], [6] and neurodegenerative disease such as Alzheimer's disease [7]. Serum levels of B_12_ and folate are in addition to nutrition influenced by several biological processes including absorption, transportation and cellular uptake, as well as processing of precursors into active molecules. Heritability, utilizing di- and monozygotic twins, is estimated to be 59% and 56% for B_12_ and folate levels, respectively, indicating that there is a substantial genetic component to the population diversity in these physiological variables [8]. Identification of sequence variants that affect circulating levels of B_12_ and folate can thus give insights into the interplay of diet, genetics and human health. Genome-wide association studies (GWAS) have yielded some sequence variants influencing B_12_ levels [9]–[12], but have been less successful in identifying variants affecting folate levels [10], [11]. Thus, genome-wide significant associations with serum B_12_ levels have been convincingly reported for four loci, *FUT2*, *MUT*, *CUBN* and *TCN1* in European populations [9]–[11] and additional four loci, *MS4A3*, *CLYBL*, *FUT6* and 5q32 in a Chinese population [12]. No genome-wide significant GWAS associations have been reported for serum folate levels, however, significant association with the *MTHFR* A222V variant was demonstrated prior to the GWAS era [13], [14] and suggestive associations have been reported in European populations for two loci (*FIGN* and *PRICKLE2*) [10], [11].

The classic GWAS applied commercial chip-based genotyping and imputation of HapMap variants of which a majority were common single nucleotide variants (SNVs) with very few rare variants with minor allele frequency (MAF) <1% [15], [16]. However, the search for the truly associated functional variants and the targeted gene at each locus has been hindered by the lack of coverage of the full spectrum of the sequence variation of the human genome. Recently, focus has turned to the use of next generation sequencing of whole genomes (WGS) [17], exomes (WES) [18] or specific targets [19], all contributing to a better understanding of the spectrum of allelic variations in the human genome. We expect that attempts to directly cover low frequency and rare sequence variants through next generation sequencing, in addition to the common variants, will improve the search for functional variants and thus the understanding of the underlying biology of human traits and diseases.

Here we aimed to identify and characterize associations of SNVs across the allele frequency spectrum with serum levels of B_12_ and folate by compiling data in up to 45,576 individuals based on sequencing initiatives in Iceland and Denmark. For the first time we apply next generation sequence data to identify sequence variants affecting serum levels of B_12_ and folate and the present datasets are the largest utilized to date for the analysis of these traits.

## Results

### Heritability of serum B_12_ and folate levels

We estimated the heritability of B_12_ and folate serum levels based on 38,229 and 21,708 Icelandic sibling pairs, respectively. Our analysis revealed estimates of 27% for B_12_ and 17% for folate which are lower than previously reported [8].

### Experimental design

**Figure 1:**
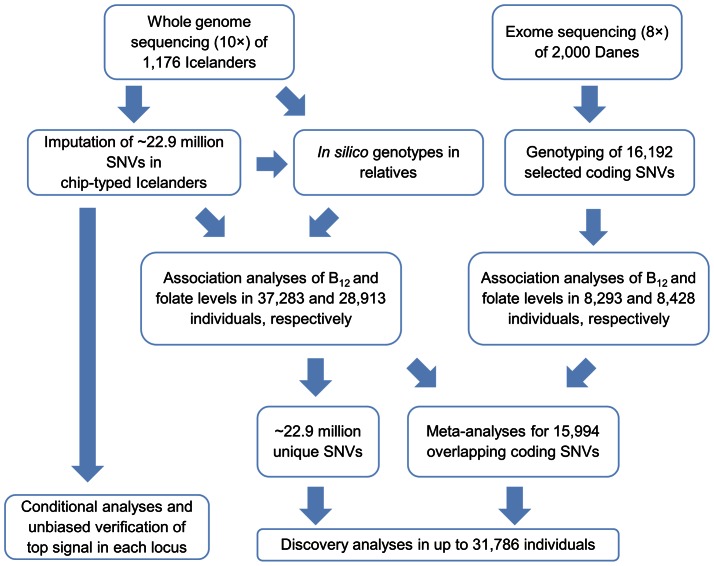
Schematic overview of the study.

To search for sequence variants affecting serum B_12_ and folate levels we compiled data from two sequencing initiatives in Iceland and Denmark. In Iceland, a large population-based resource has been generated applying WGS and highly accurate imputation of the sequence information into a large fraction of the population [20], [21]. Utilizing this resource many low frequency and rare causative sequence variants have recently been discovered that affect the risk of common diseases [22]–[26]. In the Danish samples, WES was used to search for low frequency variation associated with complex traits [27], [28]. The outline of the present study is depicted in Figure 1. In the Icelandic study sample, 1,176 individuals were whole genome sequenced to an average depth of >10× and 22.9 million SNVs were identified. These variants were then imputed into 25,960 and 20,717 chip-genotyped Icelanders with serum B_12_ and folate measurement, respectively, using highly accurate long-range phasing based imputation [20]. The Icelandic genealogical database allowed for further propagation of the sequence information, applying genealogy based imputation, into 11,323 and 8,196 relatives of the chip-genotyped individuals, for a total sample size of 37,283 and 28,913, respectively, for the two phenotypes [25] (Text S1 and Table S1). In the Danish part of the study whole exomes of 2,000 Danes were sequenced to an average sequencing depth of 8× [28]. From that effort, 16,192 coding SNVs with allelic frequency above 1% were selected for Illumina iSelect genotyping in two Danish population-based cohorts of 8,293 individuals with measurements of serum B_12_ and 8,428 individuals with measurement of serum folate (Table S2). Of the 16,192 SNVs, 15,994 overlapped with the Icelandic variants.

A generalized form of linear regression was used to test for association of serum levels of B_12_ or folate with SNVs, taking into account relatedness and population stratification within each sample set, applying the method of genomic control (GC). Analyses were performed in three steps; sequence variants were analyzed in the Icelandic and Danish samples separately, then by combining in a meta-analysis the overlapping sequence variants identified in both study samples. Loci that associated significantly with B_12_ or folate levels from these studies were fine mapped using the Icelandic WGS data imputed into chip genotyped individuals and the same data set was used to identify additional signals at each of these loci trough conditional analysis. Finally, the full Icelandic data of 22.9 million SNVs were used in GWAS to identify additional loci represented by non-coding variants or rare coding signals not genotyped in the Danish design. Genome-wide significance (GWS) level in the study was set at *P*<2.2×10^−9^, based on Bonferroni correction for the 22.9 million SNVs (Figure 1).

### Discovery analyses for serum B_12_ and folate

In the separate and combined analyses of SNVs with serum B_12_ and serum folate levels in the Icelandic and Danish data, a total of 13 genetic loci were found to associate at GWS, *P*<2.2×10^−9^ (Table 1 and 2, Figure S1 and S2). Of the 11 loci associated with serum B_12_, five (*CD320*, *TCN2*, *ABCD4*, *MMAA* and *MMACHC*) were novel and six were previously reported either in populations of European or East-Asian ancestry [9]–[12] (Table 1). Association analyses with serum folate yielded one novel locus (*FOLR3*) and confirmed the reported *MTHFR* locus (Table 2).

**Table 1:** Novel and previously reported genomic loci that associate with serum B_12_ levels at *P*<2.2×10^−9^.

SNV name	Locus	Chr.	Position (build 36/hg18)	Annotation^1^	Alleles^2^ (effect/other)	EAF	Icelandic		Danish – Inter99		Danish – Health2006		Combined		
							Effect	*P*	Effect	*P*	Effect	*P*	N	*P*	*I* 2 (*P*_HET_)
*Novel loci*															
rs2336573	*CD320*	19	8,273,709	G220R	T/**C**	0.031	0.32	1.1×10^−51^	0.22	0.0057	0.31	1.7×10^−8^	45,575	8.4×10^−59^	41 (0.033)
rs1131603	*TCN2*	22	29,348,975	L376S	C/**T**	0.055	0.19	4.3×10^−28^	0.33	1.8×10^−9^	0.33	5.3×10^−17^	45,575	4.9×10^−49^	62 (0.0050)
rs3742801	*ABCD4*	14	73,828,759	E368K	T/**C**	0.294	0.045	5.3×10^−8^	0.093	7.6×10^−4^	0.083	4.5×10^−5^	45,571	1.7×10^−13^	0 (0.20)
rs2270655	*MMAA*	4	146,795,868	Q363H	**G**/C	0.941	0.066	3.5×10^−5^	0.30	2.8×10^−7^	0.25	5.8×10^−8^	45,576	2.2×10^−13^	79 (7.1×10^−5^)
rs12272669	*MMACHC*	1	45,747,242	R206Q	A/**G**	0.0022	0.51	3.0×10^−9^	-	-	-	-	-	-	-
*Novel SNV associations in reported loci*															
rs34324219	*TCN1*	11	59,379,954	D301Y	**C**/A	0.881	0.21	8.8×10^−71^	0.40	3.2×10^−23^	0.30	3.5×10^−24^	45,576	1.1×10^−111^	70 (0.001)
rs7788053^3^	*FUT6*	19	5,783,209	P124S	A/**G**	0.254	0.046	2.1×10^−7^	0.050	0.076	0.070	0.00072	45,575	1.7×10^−10^	0 (0.64)
*Reported associated SNVs*															
rs602662	*FUT2*	19	53,898,797	G258S	A/**G**	0.596	0.16	4.1×10^−96^	0.19	3.5×10^−13^	0.23	1.9×10^−34^	45,568	2.4×10^−139^	0 (0.14)
rs1801222	*CUBN*	10	17,196,157	F253S	G/**A**	0.593	0.11	1.1×10^−52^	0.14	7.6×10^−8^	0.17	2.9×10^−18^	45,576	3.3×10^−75^	0 (0.48)
rs41281112	*CLYBL*	13	99,316,635	R259X	**C**/T	0.948	0.17	9.6×10^−27^	0.24	0.0013	0.29	2.5×10^−7^	45,576	8.9×10^−35^	0 (0.90)
rs1141321^4^	*MUT*	6	49,520,392	R532H	**C**/T	0.627	0.061	1.4×10^−16^	0.12 3	1.4×10^−5^	0.11	1.4×10^−7^	45,574	3.6×10^−26^	0 (0.24)

Association results for serum B_12_ in Icelandic and Danish study samples separately and combined. The effect allele is the allele associated with increased serum B_12_ levels. The effect is on a quantile normalized scale. Data were combined in fixed effect meta-analyses based on *P*-value and direction of effect adjusted for the number of individuals in each sample. Values of *I* ^2^ are percentages.

Chr., chromosome; EAF, effect allele frequency; HET, heterogeneity; SNV, single nucleotide variant.

1 The annotation is based on the RefSeq hg18.

2 The reference alleles based on Build 36 hg18 are shown in bold.

3 In the Icelandic data the strongest signal at the *FUT6* locus is for rs708686 located 5′ to the *FUT6* gene (see Table S3).

4 Danish data are given for the perfect proxy rs4267943 (1000 Genomes data: *r* ^2^ = 1.0).

**Table 2:** Novel and previously reported genomic loci that associate with serum folate levels at *P*<2.2×10^−9^.

SNV name	Locus	Chr.	Position (build 36)	Annotation^1^	Alleles^2^ (effect/other)	EAF	Icelandic		Danish – Inter99		Danish – Health2006		Combined		
							Effect	*P*	Effect	*P*	Effect	*P*	N	*P*	*I* 2 (*P*_HET_)
*Novel loci*															
rs652197	*FOLR3* 3 *^,^* 4	11	71,527,389	Intron	**C**/T	0.179	0.069	2.5×10^−10^	0.066	0.011	0.071	0.043	37,465	1.4×10^−12^	0 (0.77)
*Reported locus*															
rs1801133	*MTHFR*	1	11,778,965	A222V	**G**/A	0.668	0.096	1.0×10^−28^	0.18	1.1×10^−10^	0.18	5,9×10^−19^	37,337	9.5×10^−53^	62 (0.005)

Association results for serum folate in Icelandic and Danish study samples separately and combined. The effect allele is the allele associated with increased serum folate levels. The effect is on a quantile normalized scale. Data were combined in fixed effect meta-analyses based on *P*-value and direction of effect adjusted for the number of individuals in each sample. Values of *I* ^2^ are percentages. Association between serum folate levels and *MTHFR* rs1801133 in the Inter99 cohort has been published previously [14]. Chr., chromosome; EAF, effect allele frequency; HET, heterogeneity; SNV, single nucleotide variant.

1 The annotation is based on the RefSeq hg18.

2 The reference allele based on Build 36 hg18 is shown in bold.

3 In the Icelandic data a 2 bp INDEL in exon 3 of *FOLR3* associated more strongly with serum folate levels. As only SNVs were analyzed in the Danish data this data was not available for the Danish samples.

4 The rs652197 variant was initially discovered in the Icelandic samples but subsequently genotyped in Danish samples to confirm the association.

Since only coding variants were in the combined analysis we used the Icelandic WGS-based data to screen for stronger non-coding signals at the loci identified in meta-analysis of coding variants. Interestingly, the strongest signal at 10 of the 11 B_12_-associated loci in the Icelandic data corresponded to missense (*n* = 9) or nonsense (*n* = 1) mutations with only the *FUT6* locus having a stronger non-coding signal (rs708686) than the missense P124S mutation (Table S3). As only SNVs had been called from the WGS data and imputed into the Icelandic samples we reassessed each of the 13 B_12_ and folate loci with INDEL data called using the GATK algorithm (http://www.broadinstitute.org/gatk/). None of the INDELs detected at the 11 B_12_ loci associated more strongly than the lead SNVs. However, when reassessing each of the two folate-associated loci we detected a two nucleotide insertion (rs139130389, NM_000804:exon3:c.318_319insTA) encoding a common (MAF 10.0%) frameshift mutation in exon 3 of *FOLR3*, that associated more strongly with folate levels than the intronic SNV rs652197 identified in the initial scan (rs139130389: *P* = 2.45×10^−12^; effect = 0.087 SD, Table 2). The insertion and rs652197 are in linkage disequilibrium (LD) in the Icelandic sequencing data (*r* ^2^ = 0.51). Upon further inspection, we found that the ancestral sequence contained the insertion indicating the occurrence of a two base deletion in humans. The deletion with an allelic frequency of 90% in Iceland creates a premature stop codon at amino acid position 107 compared to the full-length protein consisting of 245 amino acids. Coding variants are thus lead signal of both folate loci (*FOLR3* and *MTHFR*).

The lead SNVs included both rare, low frequency and common variants with MAFs ranging from 0.2% to 48% (Table 1 and 2). Of the six novel loci, four contained a lead variant with MAF below 6% with the rare missense rs12272669 variant (MAF 0.22%) in *MMACHC* that associates with B_12_ found in the Icelandic data being at the extreme (Table 1). This variant has been observed in other populations than the Icelandic, albeit at much lower frequency (MAF 0.02%) (Exome Variant Server, http://evs.gs.washington.edu/EVS/). For *TCN1* and *FUT6* previously reported to associate with serum B_12_ levels we confirmed the association, yet with different SNVs than reported. At the *TCN1* locus the strongest associated SNV in the Icelandic data was rs34324219 (Table 1) encoding a D301Y missense mutation, whereas the reported [10], [11] and correlated (*r* ^2^ = 0.28) non-coding rs526934 was more weakly associated (Table S4). At the *FUT6* locus, the P124S missense mutation (rs778805) identified in the combined analysis of Icelandic and Danish data associated more strongly (Table 1) than the previously reported promoter rs3760776 variant (Table S4). For the remaining four reported B_12_-associated loci, *MUT*, *FUT2*, *CUBN* and *CLYBL*, we confirmed the association signal [9]–[11] (Table 1). At the *MTHFR* locus the strongest folate association was for the major allele of the common A222V (rs1801133) for which previous association with serum folate has been reported [10], [13], [14] (Table 2).

For the two loci reported to associate with B_12_ levels in individuals of East-Asian ancestry (*MSRA* and *5q32*) the variant was either not present in the Icelandic data or at very low frequency (Table S4) whereas the reported non-coding folate signals at *FIGN* and *PRICKLE2* loci did not replicate in the Icelandic folate data (Table S5).

At a less stringent significance level of *P*<1×10^−6^ we found three additional loci, *CPS1*, *SPACA1* and *ZBTB10* with suggestive associations with serum B_12_ levels (Table S6) while suggestive association with folate levels at *P*<1×10^−6^ was found for eight additional loci (Table S7).

### Analyses conditional on the identified associated sequence variants

**Figure 2:**
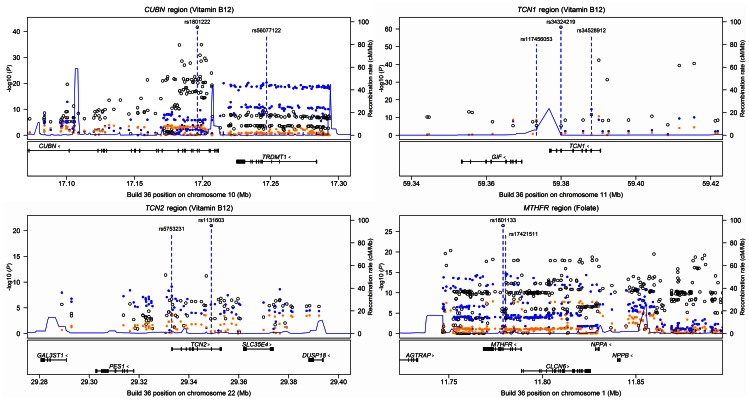
Regional plots illustrating conditional analyses of loci with more than one independent association signal for serum B_12_ (*CUBN*, *TCN1* and *TCN2*) or serum folate (*MTHFR*). Genotyped and imputed SNVs passing quality control measures in the Icelandic data are plotted with their *P*-values (as −log10 values) as a function of genomic position (NCBI Build 36). Only SNVs with *P*<10^−5^ in at least one of the models are shown. The analyses were performed in 25,960 and 20,717 chip-genotyped Icelanders for B_12_ and folate, respectively. Data points illustrated by open circles represent unconditional analyses (M0); blue dots are results of analyses conditional on the most significant SNV in M0 (M1) and orange dots are results of analyses conditional on most significant SNVs in M0 and M1. Estimated recombination rates (HapMap CEU) are plotted to reflect the local LD structure. Gene annotations were obtained from RefGene.

For the 13 loci associated with serum B_12_ or folate levels we performed stepwise conditional analyses to search for secondary signals applying Icelandic WGS data imputed into the 25,960 and 20,717 chip-genotyped Icelanders with serum B_12_ and folate information. We detected additional signals at five loci, *CUBN*, *TCN1*, *TCN2*, *FUT6* and *MTHFR* (Figure 2). For the serum B_12_-associated loci, secondary independent association signals at *P*<5×10^−8^ were detected at three, *CUBN*, *TCN1* and *TCN2* (Figure 2, Table 3, Table S8), while the secondary independent signal at *FUT6* (observed for the reported B_12_-associated rs3760776 upstream of *FUT6* [12]) did not reach the threshold of significance (*P* = 4.4×10^−6^). The secondary signal at the *CUBN* locus was shown for a group of correlated markers represented by rs56077122 (located in an intron of the neighboring *TRDMT1*) (Figure 2). In *TCN1* two additional independent signals at *P*<5×10^−8^ for serum B_12_ were found including a missense variant (R35H) and an intergenic variant whereas one secondary signal in the *TCN2* locus, represented by rs5753231, was located immediately 5′ to *TCN2* (Figure 2, Table 3). In the folate-associated loci, a secondary independent signal was found at the *MTHFR* locus represented by rs17421511 located in intron 4 of the *MTHFR* gene (Figure 2, Table 3). In contrast to the lead SNVs a large fraction of the secondary B_12_ or folate signals were non-coding.

**Table 3:** Novel secondary association signals at the serum B_12_ or serum folate loci that associate at *P*<5×10^−8^.

SNV #	SNV name	Chr.	Position (build 36)	Gene	Annotation	Alleles (effect/other)	EAF	Effect	*P*	LD with SNV #1 (*r* ^2^)	LD with SNV #2 (*r* ^2^)
***CUBN*** ** region (B_12_)**											
1	rs1801222	10	17,196,157	*CUBN*	F253S	G/A	0.593	0.11	2.3×10^−42^		
2	rs56077122	10	17,247,021	*TRDMT1*	intronic	A/C	0.335	0.087	4.8×10^−21^	0.033	
***TCN1*** ** region (B_12_)**											
1	rs34324219	11	59,379,954	*TCN1*	D301Y	C/A	0.889	0.21	9.8×10^−62^		
2	rs34528912	11	59,388,111	*TCN1*	R35H	T/C	0.0361	0.17	2.1×10^−15^	0.0040	
3	rs117456053	11	59,373,407	Near *TCN1*	Intergenic	G/A	0.976	0.16	1.9×10^−9^	0.0035	0.0011
***TCN2*** ** region (B_12_)**											
1	rs1131603	22	29,348,975	*TCN2*	L376S	C/T	0.055	0.17	1.1×10^−21^		
2	rs5753231	22	29,333,069	*TCN2*	5′	C/T	0.79	0.064	7.5×10^−10^	0.014	
***MTHFR*** ** region (folate)**											
1	rs1801133	1	11,778,965	*MTHFR*	A222V	G/A	0.668	0.10	3.4×10^−27^		
2	rs17421511	1	11,780,375	*MTHFR*	Intronic	G/A	0.827	0.098	1.8×10^−15^	0.11	

Conditional analyses were performed using imputed sequence data from chip-genotyped Icelanders with information on serum B_12_ or folate levels. Results for SNV #1 (lead SNVs) at each loci are unconditional on other SNVs. Analysis of SNV #2 is conditional on SNV #1 and SNV #3 is conditional on SNV #1 and #2. The LD between the SNVs at each locus was estimated from the sequence information of the 1,179 whole genome sequenced Icelanders.

Of the identified variants (lead and secondary) the fraction of variance in serum B_12_ or folate levels explained is estimated to be 6.3% for B_12_ and 1.0% for folate (Text S1).

### Mapping effects of associated sequence variants on gene expression

To determine whether any of the lead or secondary association signals at the B_12_ or folate loci affect the expression of the target gene we analyzed genome-wide expression QTL (eQTL) data from white blood cells (*n* = 1,001) and adipose tissue (*n* = 673) from Icelanders with information on 22.9 million SNVs [29]. Of the lead and secondary B_12_ or folate signals that are coding (Tables 1–3) two showed strong association with the expression of the target gene; the R532H missense variant in *MUT* (*P* = 9.1×10^−59^ in white blood cells and *P* = 2.5×10^−16^ in adipose tissue) and the frameshift INDEL in *FOLR3* (*P* = 7.1×10^−110^ in white blood cells and *P* = 2.5×10^−62^ in adipose tissue; Table S9). Of all the *cis* variants at the *MUT* locus the R532H missense mutation had by far the strongest effect on *MUT* expression indicating that this effect is not mediated by a non-coding regulatory variant in LD with the R532H mutation. The large effect of the frameshift mutation on *FOLR3* expression is likely caused by nonsense-mediated decay of transcripts containing the premature termination mutation [30]. A similar effect was not seen for the nonsense mutation in the *CLYBL* gene which can likely be explained by the closeness of the mutation to the N-terminal of the CLYBL protein (amino acid 259 of 340) (Table S9). Of the non-coding lead or secondary B_12_ or folate signals a statistically significant effect on expression was only seen for the *TCN2* promoter variant, however, other markers in the region, that had no effect on serum B_12_ levels associated more strongly with *TCN2* expression. Although lack of appropriate tissue to evaluate the effect of the B_12_ and folate mutations on expression cannot be excluded, these data suggest that except for the *MUT* gene the effects of both the coding and non-coding mutations are unlikely to be through expression.

### Association of identified sequence variants with other traits linked to B_12_ and folate levels

Rare mutations in some of the B_12_ genes described here i.e. *MMACHC*, *MMAA*, *MUT*, *CD320*, *TCN2* and *CUBN* have been described in connection with rare conditions of methylmalonic aciduria and megaloblastic anemia that all relate to defects in B_12_ metabolism (OMIM database, http://www.ncbi.nlm.nih.gov/omim/). In addition, epidemiological studies have suggested a link between reduced B_12_ and folate levels and the risk of common conditions such as cardiovascular diseases [3], [4], cancers [5], [6] and neurodegenerative disorders [7]. To evaluate the effect of the B_12_ or folate variants on these conditions we analyzed the association with coronary artery disease (CAD), stroke, colon cancer, prostate cancer and Alzheimer's disease in data obtained from deCODE's phenotype database. As outlined in Table S10, variants associated with serum B_12_ or folate levels did not consistently affect the risk of the diseases tested; the B_12_ or folate increasing allele for some variants was weakly protective and for others weakly at risk, and only two loci (*CUBN* associated with CAD and *MTHFR* with stroke) were statistically significant (*P*<0.0018) but with opposite effects on these diseases. B_12_ or folate deficiencies can lead to increased serum homocysteine [2], yet of all the B_12_ or folate loci tested only two associated significantly with homocysteine levels, with the B_12_ or folate increasing allele decreasing the homocyteine levels as expected (Table S10). These loci were the folate-associated *MTHFR* variant previously reported to associate with homocysteine [10], [31], [32] and the B_12_-associated variant at the *MUT* locus. Neither of these loci associated with cardiovascular disease or Alzheimer's disease, despite increased homocysteine has been suggested to increase the risk of these diseases. Deficiency of B_12_ or folate is associated with megaloblastic anemia characterized by the presence of abnormally large red blood cells, increased mean corpuscular volume (MCV) and increased mean corpuscular hemoglobin (MCH). None of the identified variants associated significantly with MCV and MCH (Table S10). We also tested the recessive model for the B_12_ or folate variants in relation to these conditions, but did not detect any new associations. Inconsistency in the direction of the effect of each of the variants on these conditions (increased or decreased risk) (Table S10) indicates that for a given condition the combined effect of all the variants would be consistent with lack of association. The absence of observed directional consistent effects of the B_12_ and folate variants on the phenotypes tested suggest that sequence variants that contribute to the population diversity in serum B_12_ or folate levels do not modify the risk of developing these conditions, likely reflecting that B_12_ and folate levels have weak effects on these conditions. However, we recognize that for some of the conditions analyzed sample sizes are too small to detect weak effects, calling for cautious interpretation.

### Evaluation of pleiotropic effects of the identified variants

One of the B_12_-associated loci, *FUT2*, has previously been associated with reduction in liver enzymes including alkaline phosphatase (ALP) [33] and cholesterol levels [34], increased risk of Crohn's disease [35], [36], psoriasis [37], retinal vascular caliber [38] and type 1 diabetes [39] and protection against Norovirus infection [40]. These associations can be explained by the function of *FUT2* in cell surface glycobiology as determinant of the Lewis antigen blood group. To evaluate pleiotropic effects of the identified B_12_ and folate variants, we screened the deCODE phenotype database, which contains information on the majority of common diseases and their associated risk factors (*n* = 400), applying both multiplicative and recessive genetic models (*P* = 3.5×10^−6^ after Bonferroni correction). We found that the *FUT2* variant associated strongly with serum levels of ALP (*P* = 1.1×10^−73^) and also with psoriasis (*P* = 4.3×10^−3^) as previously reported. We also detected a strong association with serum levels of cancer antigen 19-9 (*P* = 1.1×10^−146^), lipase (*P* = 2.2×10^−24^) and suggestive association with bone mineral density (BMD) (*P* = 1.3×10^−5^) with the B_12_-increasing allele decreasing ALP levels, increasing the serum levels of the cancer antigen 19-9 and lipase and increasing the risk of developing low BMD (osteoporosis) (Table S11). An increase in serum lipase is associated with Crohn's disease [41], but the causal link is unclear. The increased risk for low BMD observed for the *FUT2* variant may be secondary to reduced ALP activity that might be a reflection of reduced bone remodeling. When applying the recessive model to the B_12_ and folate variants we found suggestive associations of the *FUT6* variant with abdominal aortic aneurysm (AAA) and of the folate-associated variant in *MTHFR* with thoracic aortic aneurysm (TA). In both cases the effect of the B_12_- or folate-increasing allele was protective (Table S11). These associations could be mediated through the effect of these variants on B_12_ and folate levels as reduced levels of B_12_ and folate have been linked to the development of aortic aneurysm [42].

## Discussion

**Figure 3:**
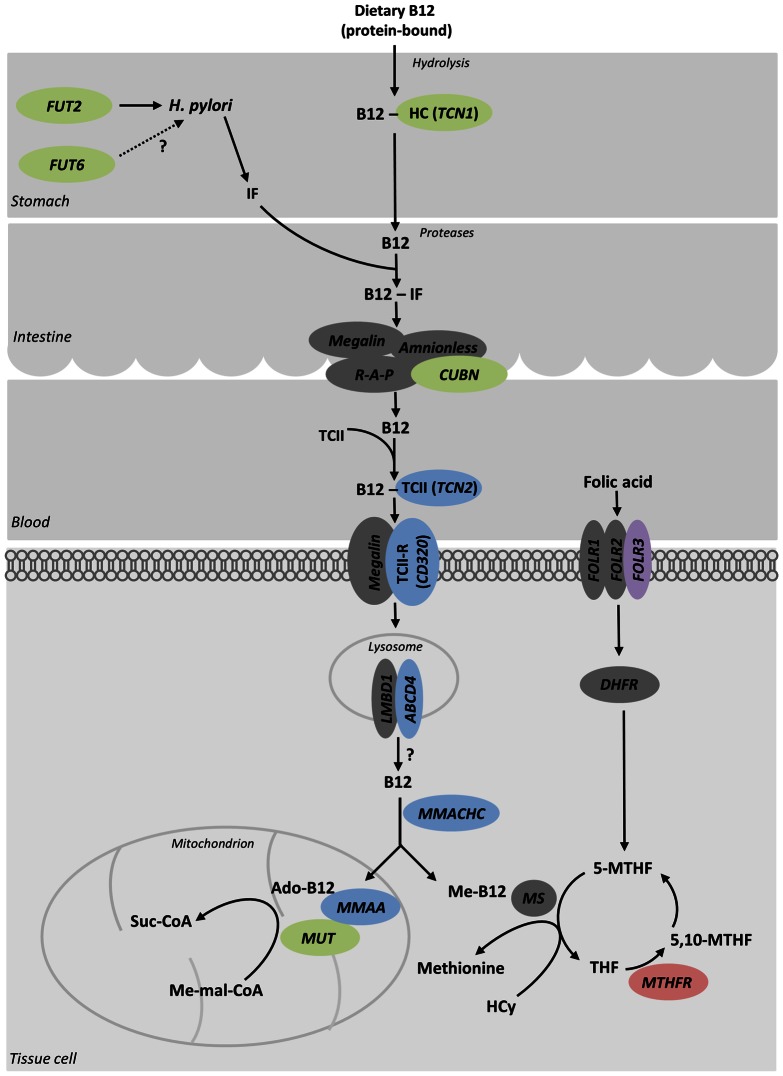
Genes that associate with serum B_12_ and folate levels are in pathways affecting their metabolism. Genes previously identified to harbor variants regulating serum levels of B_12_ are shown in green. In blue are novel genes identified in the present study. In red, genes containing variants previously suggested to associate with serum folate and in purple are novel genes for serum folate. B12: vitamin B_12_; HC: Heptocorrin (*TCN1*); IF: Intrinsic factor; R-A-P: Receptor-Associated-Protein; CUBN: cubilin (intrinsic factor-cobalamin receptor); TCII: Transcobalamin II (*TCN2*); TCII-R: Transcobalamin II receptor (*CD320*); MMACHC: methylmalonic aciduria (cobalamin deficiency) cblC type, with homocystinuria; MMAA: methylmalonic aciduria (cobalamin deficiency) cblA type; ABDC4: ATP-binding cassette, sub-family D (ALD), member 4; LMBD1: LMBR1 domain containing 1; FOLR1–3: folate receptors 1–3; Ado-B12: Adenosyl-cobalamin; Me-B12: Methyl-cobalamin; Me-mal-CoA: Methyl-malonyl-CoenzymeA; Suc-CoA: Succinyl-CoenzymeA; MUT: methylmalonyl-CoA mutase; H. pylori: Helicobacter pylori; DHFR: Dihydrofolate reductase; MS: methionin synthase; THF: Tetrahydrofolate; 5,10-MTHF: 5,10-Methyl-tetrahydrofolate; Hcy: Homocysteine MTHFR: 5-methyl-tetrahydrafolate reductase.

Here we performed association analyses of up to 22.9 million SNVs, identified through WGS and WES, in up to 45,576 individuals to identify and characterize genetic variation influencing population diversity in serum levels of B_12_ and folate. We discovered five novel loci that associate with serum B_12_ levels and one novel locus for folate levels and replicated the six reported B_12_ loci and one folate locus. In addition, we identified five novel secondary independent signals at both the new and previously reported loci. The fraction of variance in serum B_12_ or folate levels explained by the identified variants is estimated to be 6.3% for B_12_ and 1.0% for folate (Text S1). Of the identified SNVs, both common and rare, we find that a large fraction (13 of 18) is represented by coding variants which is an unusually high fraction of coding variants compared to previous GWAS for other traits. Furthermore, of the 13 loci that associate with serum B_12_ and folate levels the genes at 11 of them can be directly linked to the current understanding of B_12_ and folate metabolism such as absorption, transport or enzymatic processes and one (*FUT6*) has potential links with these processes (Figure 3). Only *CLYBL* has a function that cannot be directly related to these pathways. Specifically, eight loci are involved in transporting B_12_ and folate between different tissues, four of them *TCN1*, *FUT2*, *FUT6* and *TCN2* as co-factors or regulators of co-factors necessary for the transport and the other four, *CUBN*, *CD320*, *ABCD4* [43] and *FOLR3* as membrane transporters actively facilitating membrane crossing. *MUT* and *MTHFR* catalyze enzymatic reactions in the OCM where *MMACHC* and *MMAA* are involved in co-enzymatic processes (Figure 3). Moreover, we note that of the 13 genes, two (*TCN2* and *CD320*) are known and two (*MUT* and *MMAA*) are suggested to interact *in vivo* [44] (Figure 3). Together with the high fraction of coding mutations these data indicate that the target genes at all of the loci have been identified.

By screening the deCODE database for pleiotropic effects of the B_12_ and folate variants we replicated some of the previous associations of the *FUT2* gene and detected novel suggestive association with increased risk of osteoporosis (low BMD) potentially mediated through diminished bone remodeling as a consequence of reduced ALP activity. We also detected suggestive associations of the *FUT6* and the *MTHFR* variants with AAA and TA, respectively. However, we did not demonstrate association of any of the variants with the cardiovascular diseases, CAD and stroke, colorectal cancer, prostate cancer or Alzheimer's disease and only two of the variants associated with homocysteine levels. Although to some degree impeded by low statistical power for some of these conditions, these data suggest that sequence variants that contribute to the population diversity in serum B_12_ or folate levels do not modify the risk of developing these conditions.

## Materials and Methods

### Ethics statement

All participants gave written informed consent. The studies were conducted in accordance with the Declaration of Helsinki II and were approved by the local Ethical Committees (approval numbers Denmark: H-3-2012-155, KA 98155 and KA-20060011, DeCode 08-105-V3-S1 (issued 30.08.2011) ref. VSNb2008060006/03.1).

### Study participants in Iceland

For the Icelandic samples, serum B_12_ and folate levels were assessed in blood samples from Icelanders at the Landspitali University Hospital Laboratory or at the Icelandic Medical Center (Laeknasetrid) Laboratory in Mjodd (RAM), between the years 1990 and 2011. B_12_ and folate levels were normalized to a standard normal distribution using quantile normalization and then adjusted for sex, year of birth and age at measurement. For individuals for which more than one measurement was available we used the average of the normalized value.

### Study participants in Denmark

The Danish data were generated in two population-based study samples recruited in Copenhagen. The Inter99 cohort is a randomized, non-pharmacological intervention study for the prevention of ischaemic heart disease, conducted on 6,784 randomly ascertained participants aged 30 to 60 years at the Research Centre for Prevention and Health in Glostrup, Denmark [45] (ClinicalTrials.gov: NCT00289237). Detailed characteristics of Inter99 have been published previously [45]–[47]. The Inter99 cohort included 5,481 and 5,624 individuals with genotypes and measurement of serum B_12_ and folate, respectively. Health2006 is a population-based epidemiological study of general health, diabetes and cardiovascular disease of 3,471 individuals aged 18–74 years [48]. Health2006 was also conducted at the Research Centre for Prevention and Health in Glostrup, Denmark. The Health2006 cohort included 2,812 and 2,804 individuals with valid genotypes and measurement of serum B_12_ and folate, respectively. In Inter99 serum B_12_ and folate were measured by a competitive chemiluminescent enzyme immunoassay (Immulite 2000 System; Siemens Medical Solutions Diagnostics, Los Angeles, CA, USA) as previously reported [14]. In Health2006, serum B_12_ and folate were measured by chemiluminescent immunoassay (Dimension Vista platform, Siemens Healthcare Diagnostics GmbH, Eschborn, Germany).

### Genotype data generation

In the Icelandic part, SNVs were identified through the Icelandic WGS project. A total of 1,176 Icelanders were selected for sequencing based on having various neoplasic, cardiovascular and psychiatric conditions. All of the individuals were sequenced to a depth of at least 10×. The generation of genotypic data in Iceland is detailed in earlier reports [23] and in Text S1, and consisted of the following steps: SNV calling and genotyping in WGS, long range phasing, genotype imputation and *in silico* genotyping.

In the Danish part of the study 16,192 SNVs for genotyping were selected from a WES study of 2,000 individuals [28]. In brief, exon capture and Illumina sequencing to a depth of 8× were performed in 2,000 Danes by methods previously described [27]. The exome was captured by a NimbleGen 2.1M HD array with a target region of 34.1 Mb including 18,954 genes defined by CCDS (Consensus Coding Sequence database). The average number of reads sequenced for each individual was 22.3 million with most reads being 30 to 80 bases long. After alignment to the human reference genome (assembly hg18, NCBI build 36.3) and stringent quality assurance, including uniqueness of genomic mapping and Q-score >20, the median coverage per individual was 91% of the target region and had an average depth of 8× (96% coverage and 11× depth before filtering). After applying quality criteria 70,182 SNVs with an estimated MAF above 1% based on the reads using maximum likelihood were identified [49]. The details of the WES have been described previously [28]. 20,005 SNVs were, as part of a published study, selected from the exome sequencing for genotyping in 16,888 samples by a custom-designed Illumina iSelect array. First, 18,358 SNVs annotated to the most likely deleterious categories (179 nonsense, 15,789 nonsynonymous, 219 located in splice sites and 2,171 in untranslated regions) were prioritized. Second, 1,048 SNVs nominally associated with type 2 diabetes (*P*<0.05) in a sequencing-based association study were selected. Finally, we selected 599 synonymous variants in 192 loci previously associated with common metabolic traits at GWS. Genotype data was obtained for 18,744 SNVs. Quality control of samples included removing closely related individuals, individuals with an extreme inbreeding coefficient, individuals with a low call rate, individuals with a mislabeled sex and individuals with a high discordance rate to previously genotyped SNVs. 15,989 individuals passed all quality control criteria. The SNVs were filtered based on their MAF (>0.5%), genotype call rate (>95%), Hardy-Weinberg equilibrium (*P*>10^−7^) or cross-hybridization with the X-chromosome. 16,192 SNVs passed all filters [28]. Genotyping of *FOLR3* rs652197 in Danish samples was done by KASPar SNP Genotyping System (KBioscience, Hoddesdon, UK).

### Statistical analyses

#### Icelandic analyses and quantitative trait association testing

A generalized form of linear regression was used to test for association of serum B_12_ and folate with SNVs. Let 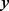 be the vector of quantitative measurements, and let 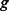 be the vector of expected allele counts for the SNV being tested. We assume the quantitative measurements follow a normal distribution with a mean that depends linearly on the expected allele at the SNV and a variance covariance matrix proportional to the kinship matrix:
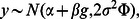
where
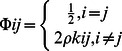
is based on the kinship between individuals as estimated from the Icelandic genealogical database 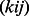 and estimate of the heritability of the trait 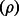. It is not computationally feasible to use this full model and we therefore split the individuals with *in silico* genotypes and serum B_12_ and folate measurements into smaller clusters. Here we chose to restrict the cluster size to at most 300 individuals.

The maximum likelihood estimates for the parameters 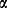, 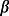, and 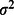 involve inverting the kinship matrix. If there are 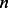 individuals in the cluster, then this inversion requires 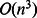 calculations, but since these calculations only need to be performed once the computational cost of doing a GWAS will only be 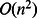 calculations; the cost of calculating the maximum likelihood estimates if the kinship matrix has already been inverted.

#### Multivariate regression and conditional analyses

For the multivariate regression analysis we only used Icelandic individuals which have been genotyped using the Illumina chip-genotyping platform. The multivariate linear regression analysis was performed conditioning for a given marker by adjusting for the estimated allele count based on imputation of this marker. The GC correction factor was the same as used for the unadjusted association analysis. A forward selection multiple logistic regression model was used to further define the extent of the genetic association. Briefly, all imputed SNVs located within an interval around the lead SNVs were tested for possible incorporation into a multiple regression model. In a stepwise fashion, a SNV was added to the model if it had the smallest *P*-value among all SNVs not yet included in the model and if it had a *P*<5×10^−8^. In the last step none of the SNVs remained significant at this threshold.

#### Association analyses of serum B_12_ and folate in Danish samples

Association analysis of each SNV in the Danish data was performed using linear regression assuming an additive model. Principal component analysis was performed using the covariance matrix and the first principal component and sex were included in the model as covariates. All quantitative traits were quantile normalized to a normal distribution prior to analysis. Association analyses were done using PLINK software (version 1.07, http://pngu.mgh.harvard.edu/purcell/plink/). All *P*-values were corrected by GC. Inflation factors (λ) were at acceptable levels: B_12_: Inter99: 1.027, Health2006: 1.014 and folate: Inter99: 1.024, Health2006: 1.010.

#### Meta-analyses

For all SNVs with data from more than one study sample (Icelandic, Inter99 and/or Health2006) we performed meta-analyses of summary association data where we estimated the combined effect in a fixed-effects meta-analysis using the METAL software (http://www.sph.umich.edu/csg/abecasis/Metal/) [50]. An overall z-statistic relative to each reference allele was estimated based on *P*-value and direction of effect adjusted for the number of individuals in each sample.

## Supporting Information

Figure S1 Regional plots of the 11 loci associated with serum B_12_. Genotyped and imputed SNVs passing quality control measures are plotted with their meta-analysis *P*-values (as −log10 values) as a function of genomic position (NCBI Build 36). Only SNVs with *P*<0.01 are plotted. The lead SNV with the lowest combined *P*-value is indicated by the rs-number. Estimated recombination rates (HapMap CEU) are plotted to reflect the local LD structure. Gene annotations were obtained from RefGene.
(PDF)

Figure S2 Regional plots of the two loci associated with serum folate. Genotyped and imputed SNVs passing quality control measures are plotted with their meta-analysis *P*-values (as −log10 values) as a function of genomic position (NCBI Build 36). Only SNVs with *P*<0.01 are plotted. The lead SNV with the lowest combined *P*-value is indicated by the rs-number. Estimated recombination rates (HapMap CEU) are plotted to reflect the local LD structure. Gene annotations were obtained from RefGene.
(PDF)

Table S1 Clinical characteristics of the Icelandic samples. Data are mean ± standard deviation or median (interquartile range). For individuals for which more than one measurement was available we used the average of the normalized value.
(PDF)

Table S2 Clinical characteristics of the Danish samples. Data are mean ± standard deviation or median (interquartile range).
(PDF)

Table S3 Overview of the most significantly associated SNV for each of the identified B_12_ or folate loci in the Icelandic data. For each of the identified B_12_ or folate loci presented in Tables 1 and 2 the Icelandic association data for the lead SNV is shown. Moreover, the strongest associations at these loci in the Icelandic data are shown. The lead SNVs presented in Tables 1 and 2 are either the strongest signal at each of the loci or highly correlated with the strongest signal except at the *FUT6* locus were rs708686 located 5′ of *FUT6* gives the strongest signal.
(PDF)

Table S4 Association results in the Icelandic data for SNVs previously reported to associate with B_12_ levels in GWAS. *These markers are only present in East-Asia. References: 1. Lin X, Lu D, Gao Y, Tao S, Yang X, et al. (2012) Genome-wide association study identifies novel loci associated with serum level of vitamin B12 in Chinese men. Hum Mol Genet 21: 2610–2617. 2. Hazra A, Kraft P, Lazarus R, Chen C, Chanock SJ, et al. (2009) Genome-wide significant predictors of metabolites in the one-carbon metabolism pathway. Hum Mol Genet 18: 4677–4687. 3. Hazra A, Kraft P, Selhub J, Giovannucci EL, Thomas G, et al. (2008) Common variants of *FUT2* are associated with plasma vitamin B12 levels. Nat Genet 40: 1160–1162.
(PDF)

Table S5 Association results in Icelandic data for SNVs previously reported with suggestive association with folate levels in GWAS. * Not previously shown at genome-wide significance. References: 1. Tanaka T, Scheet P, Giusti B, Bandinelli S, Piras MG, et al. (2009) Genome-wide association study of vitamin B6, vitamin B12, folate, and homocysteine blood concentrations. Am J Hum Genet 84: 477–482. 2. Hazra A, Kraft P, Lazarus R, Chen C, Chanock SJ, et al. (2009) Genome-wide significant predictors of metabolites in the one-carbon metabolism pathway. Hum Mol Genet 18: 4677–4687.
(PDF)

Table S6 Suggestive loci in the Icelandic or the Icelandic and Danish data associated with serum B_12_ levels (2.2×10^−9^> *P*<10^−6^).
(PDF)

Table S7 Results in the Icelandic data for loci with suggestive association with serum folate levels (2.2×10^−9^>*P*<10^−6^).
(PDF)

Table S8 Results from stepwise conditional analyses using the Icelandic data at loci associated with serum B_12_ or folate levels for signals with *P*<5×10^−8^. Conditional analyses were performed using imputed sequence data from chip-genotyped Icelanders with information on serum B_12_ or folate levels. Results for SNV #1 (lead SNVs) at each loci are unconditional on other SNVs. Analysis of SNV #2 is conditional on SNV #1 and SNV #3 is conditional on SNV #1 and #2. The LD between the SNVs at each locus was estimated from the sequence information of the 1,179 whole genome sequenced Icelanders.
(PDF)

Table S9 *Cis* **-effect of the B_12_ and folate SNVs on the expression of the target gene in white blood cells and adipose tissue.** Correlation between SNVs that associate with increased B_12_ or folate and mRNA expression in blood and adipose tissue from 1,001 and 673 individuals, respectively. The correlations are tested by regression analysis adjusted, for age, sex and differential cell counts (blood only), and inverse normal transformed relative expression values on the estimated genotype dosage. ^a^No other SNV shows significantly higher correlation with the expression in adipose or blood of *MUT* than rs1141321. ^b^The INDEL chr11:71527804 is the most significant *cis* variant for *FOLR3*. ^c^For *TCN2* there are *cis* variants both in blood and adipose tissue that have stronger correlation than rs5753231 with its expression, while having little effect on B_12_ levels.
(PDF)

Table S10 **Association results for B_12_ and folate associated markers with potential co-morbid conditions in Icelanders.** ^a^ Effect size and effect allele frequency from the Icelandic population. Associations at *P*<0.001 are shown in bold. EAF, effect allele frequency; MCV, mean corpuscular volume; MCH, mean corpuscular hemoglobin.
(PDF)

Table S11 **Association results for the B_12_ and folate variants with diseases and traits in deCODE database.** Shown are the strongest association results for the folate and B_12_ variants, genome-wide significant or suggestive, with diseases and traits in deCODE's database. EAF, effect allele frequency; BMD, bone mineral density; AAA, abdominal aortic aneurysm; TA, thoracic aneurysm; ^1^The annotation is based on the RefSeq hg18, ^2^The reference alleles based on Build 36 hg18 are shown in bold, ^3^The low BMD phenotypes are defined as those BMD values that are below −1 standard deviation (SD) from the mean.
(PDF)

Text S1 **Supplementary Text S1 contains additional description of Methods.**
(PDF)

## References

[1] Scott JM (1999) Folate and vitamin B_12_ . Proc Nutr Soc 58: 441–448. 10466189 10.1017/s0029665199000580

[2] Markle HV, Greenway DC (1996) Cobalamin. Crit Rev Clin Lab Sci 33: 247–356. 8875026 10.3109/10408369609081009

[3] Clarke R, Daly L, Robinson K, Naughten E, Cahalane S, et al (1991) Hyperhomocysteinemia: An Independent Risk Factor for Vascular Disease. N Engl J Med 324: 1149–1155. 2011158 10.1056/NEJM199104253241701

[4] Stampfer MJ, Malinow MR, Willett WC, Newcomer LM, Upson B, et al (1992) A prospective study of plasma homocyst(e)ine and risk of myocardial infarction in US physicians. JAMA 268: 877–881. 1640615

[5] Bird CL, Swendseid ME, Witte JS, Shikany JM, Hunt IF, et al (1995) Red cell and plasma folate, folate consumption, and the risk of colorectal adenomatous polyps. Cancer Epidemiol Biomarkers Prev 4: 709–714. 8672986

[6] Giovannucci E, Stampfer MJ, Colditz GA, Rimm EB, Trichopoulos D, et al (1993) Folate, Methionine, and Alcohol Intake and Risk of Colorectal Adenoma. J Natl Cancer Inst 85: 875–883. 8492316 10.1093/jnci/85.11.875

[7] Clarke R (1998) Folate, vitamin B12, and serum total homocysteine levels in confirmed Alzheimer disease. Arch Neurol 55: 1449–1455. 9823829 10.1001/archneur.55.11.1449

[8] Nilsson SE, Read S, Berg S, Johansson B (2009) Heritabilities for fifteen routine biochemical values: findings in 215 Swedish twin pairs 82 years of age or older. Scand J Clin Lab Invest 69: 562–569. 19343610 10.1080/00365510902814646

[9] Hazra A, Kraft P, Selhub J, Giovannucci EL, Thomas G, et al (2008) Common variants of FUT2 are associated with plasma vitamin B12 levels. Nat Genet 40: 1160–1162. 18776911 10.1038/ng.210 PMC2673801

[10] Hazra A, Kraft P, Lazarus R, Chen C, Chanock SJ, et al (2009) Genome-wide significant predictors of metabolites in the one-carbon metabolism pathway. Hum Mol Genet 18: 4677–4687. 19744961 10.1093/hmg/ddp428 PMC2773275

[11] Tanaka T, Scheet P, Giusti B, Bandinelli S, Piras MG, et al (2009) Genome-wide association study of vitamin B6, vitamin B12, folate, and homocysteine blood concentrations. Am J Hum Genet 84: 477–482. 19303062 10.1016/j.ajhg.2009.02.011 PMC2667971

[12] Lin X, Lu D, Gao Y, Tao S, Yang X, et al (2012) Genome-wide association study identifies novel loci associated with serum level of vitamin B12 in Chinese men. Hum Mol Genet 21: 2610–2617. 22367966 10.1093/hmg/dds062

[13] Hustad S, Midttun O, Schneede J, Vollset SE, Grotmol T, et al (2007) The methylenetetrahydrofolate reductase 677C→T polymorphism as a modulator of a B vitamin network with major effects on homocysteine metabolism. Am J Hum Genet 80: 846–855. 17436239 10.1086/513520 PMC1852731

[14] Thuesen BH, Husemoen LL, Ovesen L, Jorgensen T, Fenger M, et al (2010) Lifestyle and genetic determinants of folate and vitamin B12 levels in a general adult population. Br J Nutr 103: 1195–1204. 19968891 10.1017/S0007114509992947

[15] Hindorff LA, Sethupathy P, Junkins HA, Ramos EM, Mehta JP, et al (2009) Potential etiologic and functional implications of genome-wide association loci for human diseases and traits. Proc Natl Acad Sci USA 106: 9362–9367. 19474294 10.1073/pnas.0903103106 PMC2687147

[16] International HapMap Consortium (2007) Frazer KA, Ballinger DG, Cox DR, Hinds DA, et al (2007) A second generation human haplotype map of over 3.1 million SNPs. Nature 449: 851–861. 17943122 10.1038/nature06258 PMC2689609

[17] Genomes Project Consortium (2010) Durbin RM, Abecasis GR, Altshuler DL, Auton A, et al (2010) A map of human genome variation from population-scale sequencing. Nature 467: 1061–1073. 20981092 10.1038/nature09534 PMC3042601

[18] Tennessen JA, Bigham AW, O'Connor TD, Fu W, Kenny EE, et al (2012) Evolution and functional impact of rare coding variation from deep sequencing of human exomes. Science 337: 64–69. 22604720 10.1126/science.1219240 PMC3708544

[19] Nelson MR, Wegmann D, Ehm MG, Kessner D, St JP, et al (2012) An abundance of rare functional variants in 202 drug target genes sequenced in 14,002 people. Science 337: 100–104. 22604722 10.1126/science.1217876 PMC4319976

[20] Kong A, Masson G, Frigge ML, Gylfason A, Zusmanovich P, et al (2008) Detection of sharing by descent, long-range phasing and haplotype imputation. Nat Genet 40: 1068–1075. 19165921 10.1038/ng.216 PMC4540081

[21] Kong A, Steinthorsdottir V, Masson G, Thorleifsson G, Sulem P, et al (2009) Parental origin of sequence variants associated with complex diseases. Nature 462: 868–874. 20016592 10.1038/nature08625 PMC3746295

[22] Holm H, Gudbjartsson DF, Sulem P, Masson G, Helgadottir HT, et al (2011) A rare variant in MYH6 is associated with high risk of sick sinus syndrome. Nat Genet 43: 316–320. 21378987 10.1038/ng.781 PMC3066272

[23] Sulem P, Gudbjartsson DF, Walters GB, Helgadottir HT, Helgason A, et al (2011) Identification of low-frequency variants associated with gout and serum uric acid levels. Nat Genet 43: 1127–1130. 21983786 10.1038/ng.972

[24] Gudmundsson J, Sulem P, Gudbjartsson DF, Masson G, Agnarsson BA, et al (2012) A study based on whole-genome sequencing yields a rare variant at 8q24 associated with prostate cancer. Nat Genet 44: 1326–1329. 23104005 10.1038/ng.2437 PMC3562711

[25] Rafnar T, Gudbjartsson DF, Sulem P, Jonasdottir A, Sigurdsson A, et al (2011) Mutations in BRIP1 confer high risk of ovarian cancer. Nat Genet 43: 1104–1107. 21964575 10.1038/ng.955

[26] Jonsson T, Atwal JK, Steinberg S, Snaedal J, Jonsson PV, et al (2012) A mutation in APP protects against Alzheimer's disease and age-related cognitive decline. Nature 488: 96–99. 22801501 10.1038/nature11283

[27] Li Y, Vinckenbosch N, Tian G, Huerta-Sanchez E, Jiang T, et al (2010) Resequencing of 200 human exomes identifies an excess of low-frequency non-synonymous coding variants. Nat Genet 42: 969–972. 20890277 10.1038/ng.680

[28] Albrechtsen A, Grarup N, Li Y, Sparso T, Tian G, et al (2013) Exome sequencing-driven discovery of coding polymorphisms associated with common metabolic phenotypes. Diabetologia 56: 298–310. 23160641 10.1007/s00125-012-2756-1 PMC3536959

[29] Emilsson V, Thorleifsson G, Zhang B, Leonardson AS, Zink F, et al (2008) Genetics of gene expression and its effect on disease. Nature 452: 423–428. 18344981 10.1038/nature06758

[30] Balasubramanian S, Habegger L, Frankish A, MacArthur DG, Harte R, et al (2011) Gene inactivation and its implications for annotation in the era of personal genomics. Genes Dev 25: 1–10. 21205862 10.1101/gad.1968411 PMC3012931

[31] Pare G, Chasman DI, Parker AN, Zee RR, Malarstig A, et al (2009) Novel associations of CPS1, MUT, NOX4, and DPEP1 with plasma homocysteine in a healthy population: a genome-wide evaluation of 13 974 participants in the Women's Genome Health Study. Circ Cardiovasc Genet 2: 142–150. 20031578 10.1161/CIRCGENETICS.108.829804 PMC2745176

[32] Lange LA, Croteau-Chonka DC, Marvelle AF, Qin L, Gaulton KJ, et al (2010) Genome-wide association study of homocysteine levels in Filipinos provides evidence for CPS1 in women and a stronger MTHFR effect in young adults. Hum Mol Genet 19: 2050–2058. 20154341 10.1093/hmg/ddq062 PMC2860887

[33] Chambers JC, Zhang W, Sehmi J, Li X, Wass MN, et al (2011) Genome-wide association study identifies loci influencing concentrations of liver enzymes in plasma. Nat Genet 43: 1131–1138. 22001757 10.1038/ng.970 PMC3482372

[34] Teslovich TM, Musunuru K, Smith AV, Edmondson AC, Stylianou IM, et al (2010) Biological, clinical and population relevance of 95 loci for blood lipids. Nature 466: 707–713. 20686565 10.1038/nature09270 PMC3039276

[35] Franke A, McGovern DP, Barrett JC, Wang K, Radford-Smith GL, et al (2010) Genome-wide meta-analysis increases to 71 the number of confirmed Crohn's disease susceptibility loci. Nat Genet 42: 1118–1125. 21102463 10.1038/ng.717 PMC3299551

[36] McGovern DP, Jones MR, Taylor KD, Marciante K, Yan X, et al (2010) Fucosyltransferase 2 (FUT2) non-secretor status is associated with Crohn's disease. Hum Mol Genet 19: 3468–3476. 20570966 10.1093/hmg/ddq248 PMC2916706

[37] Ellinghaus D, Ellinghaus E, Nair RP, Stuart PE, Esko T, et al (2012) Combined Analysis of Genome-wide Association Studies for Crohn Disease and Psoriasis Identifies Seven Shared Susceptibility Loci. Am J Hum Genet 90: 636–647. 22482804 10.1016/j.ajhg.2012.02.020 PMC3322238

[38] Ikram MK, Sim X, Jensen RA, Cotch MF, Hewitt AW, et al (2010) Four novel Loci (19q13, 6q24, 12q24, and 5q14) influence the microcirculation in vivo. PLoS Genet 6: e1001184. 21060863 10.1371/journal.pgen.1001184 PMC2965750

[39] Smyth DJ, Cooper JD, Howson JMM, Clarke P, Downes K, et al (2011) FUT2 Nonsecretor Status Links Type 1 Diabetes Susceptibility and Resistance to Infection. Diabetes 60: 3081–3084. 22025780 10.2337/db11-0638 PMC3198057

[40] Carlsson B, Kindberg E, Buesa J, Rydell GE, Lidón MF, et al (2009) The G428A Nonsense Mutation in FUT2 Provides Strong but Not Absolute Protection against Symptomatic GII.4 Norovirus Infection. PLoS ONE 4: e5593. 19440360 10.1371/journal.pone.0005593 PMC2680586

[41] Hegnhoj J, Hansen CP, Rannem T, Sobirk H, Andersen LB, et al (1990) Pancreatic function in Crohn's disease. Gut 31: 1076–1079. 1698692 10.1136/gut.31.9.1076 PMC1378673

[42] Warsi AA, Davies B, Morris-Stiff G, Hullin D, Lewis MH (2004) Abdominal Aortic Aneurysm and its Correlation to Plasma Homocysteine, and Vitamins. Eur J Vasc Endovasc Surg 27: 75–79. 14652841 10.1016/j.ejvs.2003.09.001

[43] Coelho D, Kim JC, Miousse IR, Fung S, du MM, et al (2012) Mutations in ABCD4 cause a new inborn error of vitamin B12 metabolism. Nat Genet 44: 1152–1155. 22922874 10.1038/ng.2386

[44] Korotkova N, Lidstrom ME (2004) MeaB is a component of the methylmalonyl-CoA mutase complex required for protection of the enzyme from inactivation. J Biol Chem 279: 13652–13658. 14734568 10.1074/jbc.M312852200

[45] Jørgensen T, Borch-Johnsen K, Thomsen TF, Ibsen H, Glumer C, et al (2003) A randomized non-pharmacological intervention study for prevention of ischaemic heart disease: Baseline results Inter99 (1). Eur J Cardiovasc Prev Rehab 10: 377–386. 10.1097/01.hjr.0000096541.30533.82 14663300

[46] Boesgaard TW, Grarup N, Jørgensen T, Borch-Johnsen K (2010) Meta-Analysis of Glucose and Insulin-Related Trait Consortium (MAGIC), (2010) et al Variants at DGKB/TMEM195, ADRA2A, GLIS3 and C2CD4B loci are associated with reduced glucose-stimulated beta cell function in middle-aged Danish people. Diabetologia 53: 1647–1655. 20419449 10.1007/s00125-010-1753-5

[47] Glümer C, Jørgensen T, Borch-Johnsen K (2003) Prevalences of diabetes and impaired glucose regulation in a Danish population: the Inter99 study. Diabetes Care 26: 2335–2340. 12882858 10.2337/diacare.26.8.2335

[48] Thyssen JP, Linneberg A, Menne T, Nielsen NH, Johansen JD (2009) The prevalence and morbidity of sensitization to fragrance mix I in the general population. Br J Dermatol 161: 95–101. 19438476 10.1111/j.1365-2133.2009.09157.x

[49] Kim SY, Lohmueller KE, Albrechtsen A, Li Y, Korneliussen T, et al (2011) Estimation of allele frequency and association mapping using next-generation sequencing data. BMC Bioinformatics 12: 231. 21663684 10.1186/1471-2105-12-231 PMC3212839

[50] Willer CJ, Li Y, Abecasis GR (2010) METAL: fast and efficient meta-analysis of genomewide association scans. Bioinformatics 26: 2190–2191. 20616382 10.1093/bioinformatics/btq340 PMC2922887

