# Supplementary figures and images for: Genetic Architecture of Vitamin B12 and Folate Levels Uncovered Applying Deeply Sequenced Large Datasets
Source: PLoS Genet. 2013 Jun 6;9(6):e1003530. doi: 10.1371/journal.pgen.1003530 (PMC3674994; doi:10.1371/journal.pgen.1003530)

Figure S1

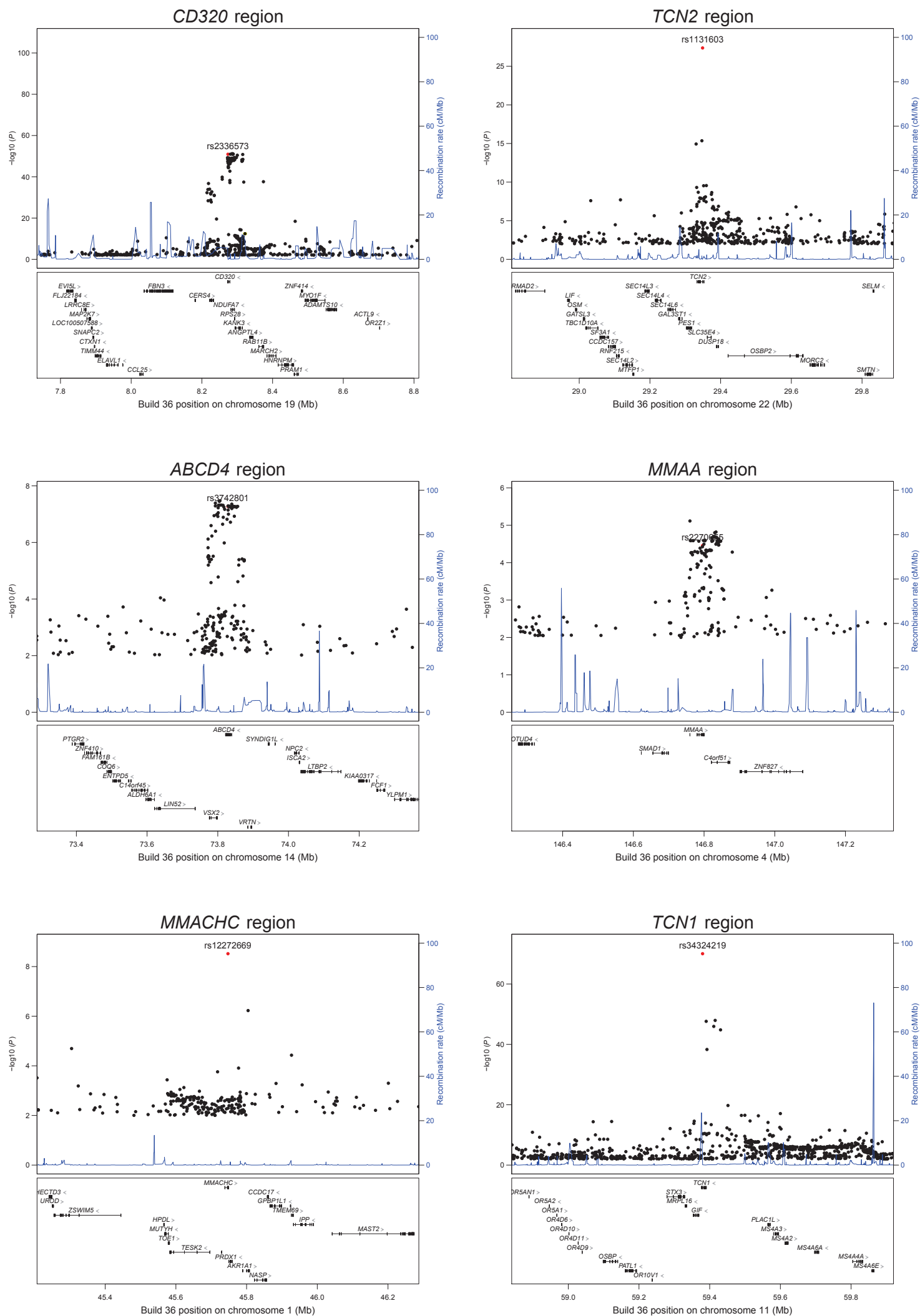

### CENPQ region

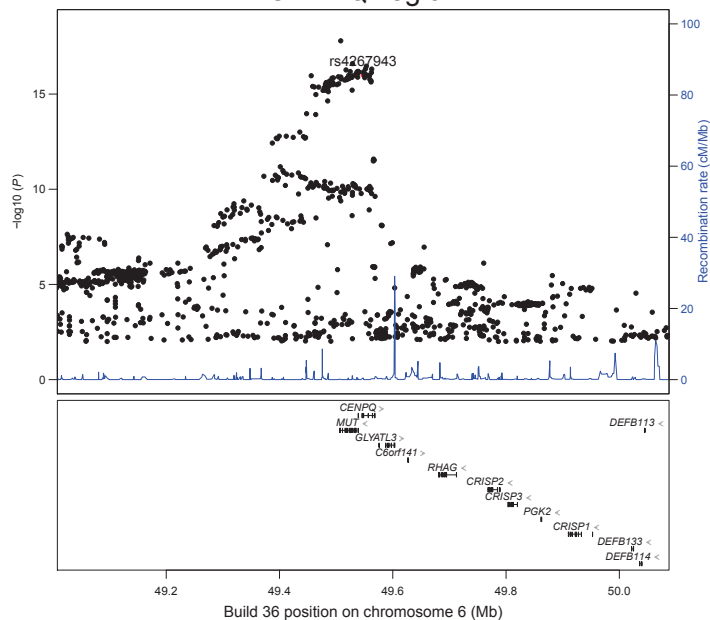

### FUT6 region

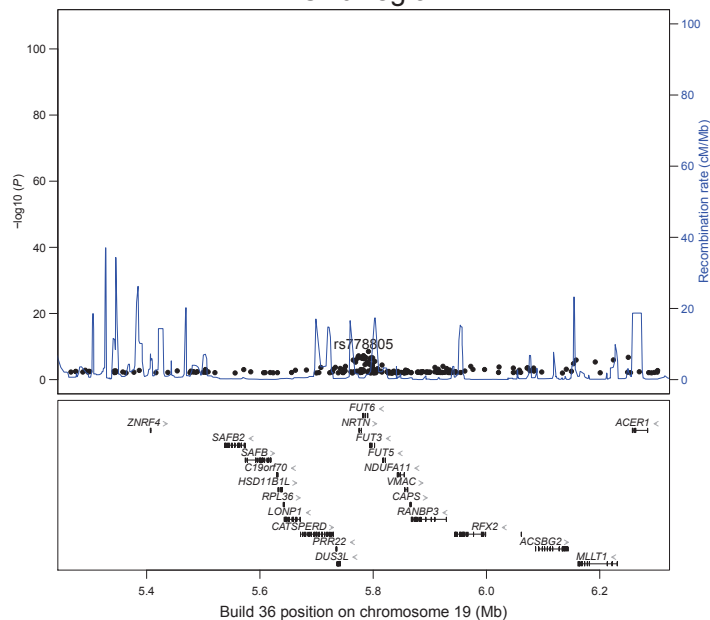

### FUT2 region

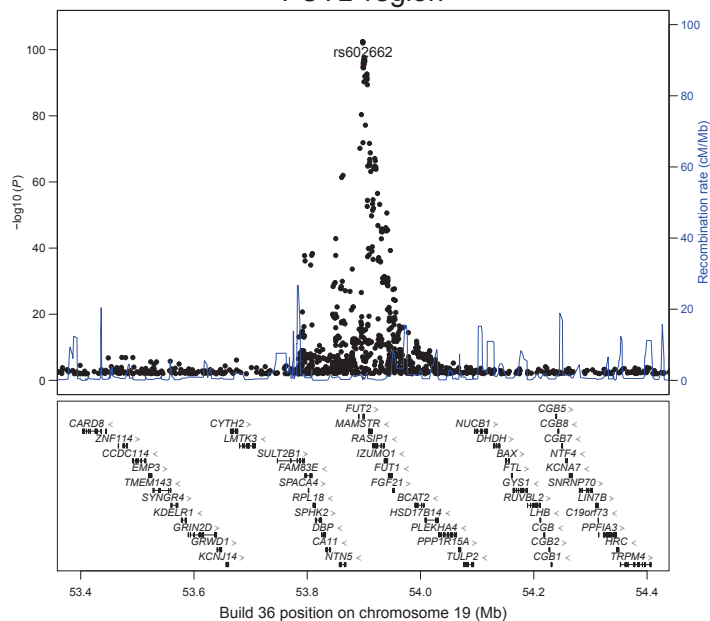

### CUBN region

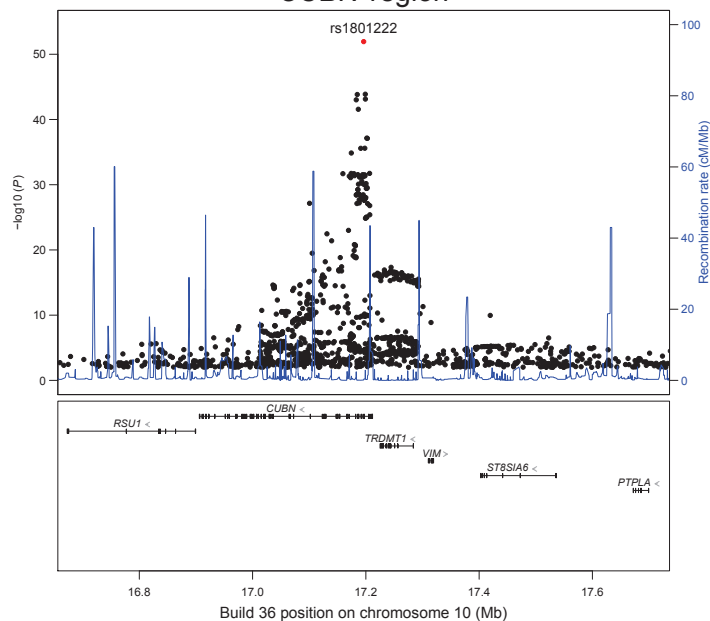

### CLYBL region

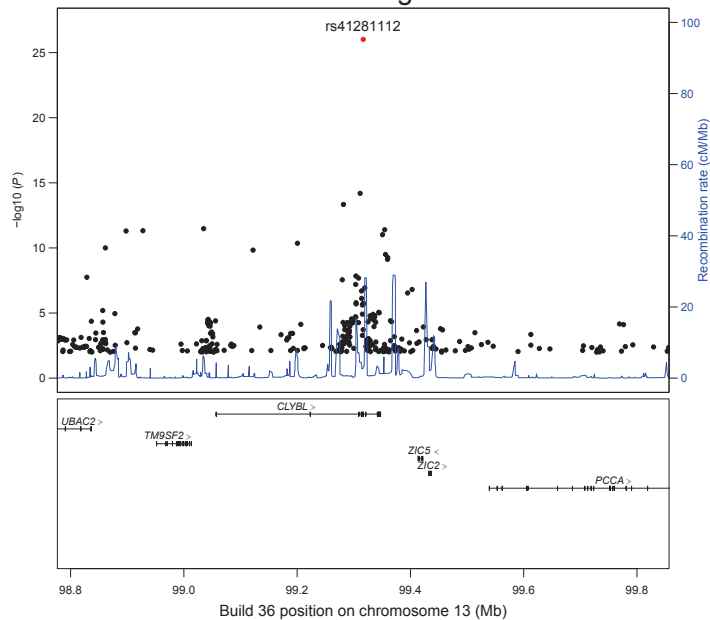

Supplement: Figure S1 — Regional plots of the 11 loci associated with serum B12. Genotyped and imputed SNVs passing quality control measures are plotted with their meta-analysis P-values (as −log10 values) as a function of genomic position (NCBI Build 36). Only SNVs with P<0.01 are plotted. The lead SNV with the lowest combined P-value is indicated by the rs-number. Estimated recombination rates (HapMap CEU) are plotted to reflect the local LD structure. Gene annotations were obtained from RefGene. (PDF) [file pgen.1003530.s001.pdf]

Figure S2

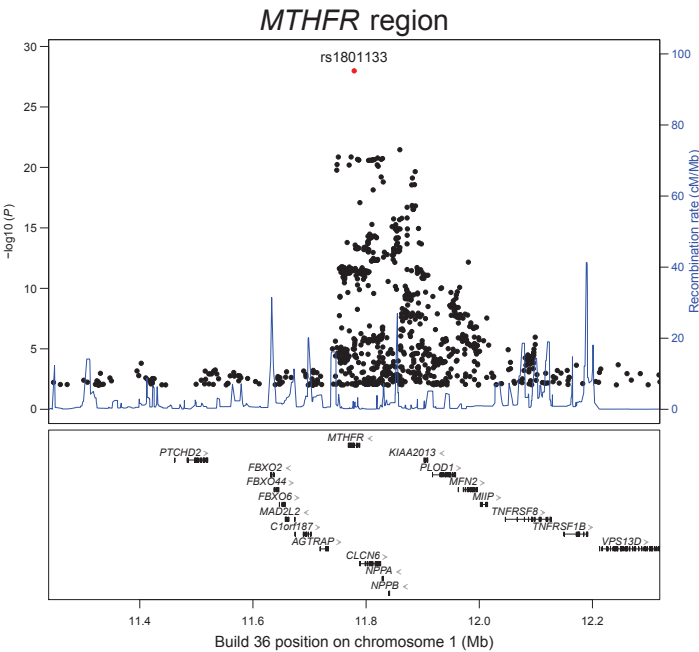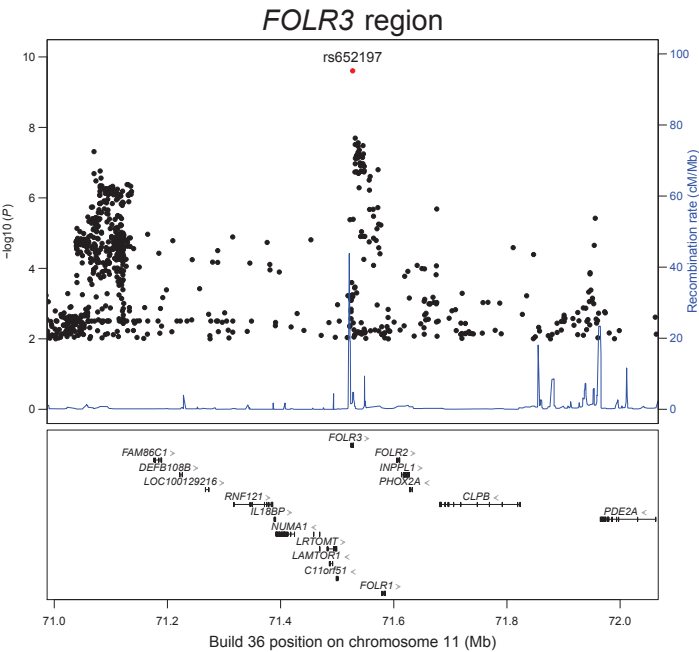

Supplement: Figure S2 — Regional plots of the two loci associated with serum folate. Genotyped and imputed SNVs passing quality control measures are plotted with their meta-analysis P-values (as −log10 values) as a function of genomic position (NCBI Build 36). Only SNVs with P<0.01 are plotted. The lead SNV with the lowest combined P-value is indicated by the rs-number. Estimated recombination rates (HapMap CEU) are plotted to reflect the local LD structure. Gene annotations were obtained from RefGene. (PDF) [file pgen.1003530.s002.pdf]
