# Supplementary material for: Genetic Architecture of Vitamin B12 and Folate Levels Uncovered Applying Deeply Sequenced Large Datasets
Source: PLoS Genet. 2013 Jun 6;9(6):e1003530. doi: 10.1371/journal.pgen.1003530 (PMC3674994; doi:10.1371/journal.pgen.1003530)
Supplement: Table S1 — Clinical characteristics of the Icelandic samples. Data are mean ± standard deviation or median (interquartile range). For individuals for which more than one measurement was available we used the average of the normalized value. (PDF) [file pgen.1003530.s003.pdf]

**Table S1.** Clinical characteristics of the Icelandic samples		
	Icelanders with serum B_{12} measurement	Icelanders with serum folate measurement
N (chip-genotyped)	25,960	20,717
N (genealogically imputed)	11,323	8,196
Men (%)	35.4	34.8
Age (yrs)	63.4 ± 23.8	63.4 ± 25.2
B_{12} (pmol/l)	409 (305-555)	-
Folate (nmol/l)	-	22.6 (15.3-38.7)
Repeats	2.6	2.3

Data are mean ± standard deviation or median (interquartile range).

For individuals for which more than one measurement was available we used the average of the normalized value.
