# Supplementary material for: Genetic Architecture of Vitamin B12 and Folate Levels Uncovered Applying Deeply Sequenced Large Datasets
Source: PLoS Genet. 2013 Jun 6;9(6):e1003530. doi: 10.1371/journal.pgen.1003530 (PMC3674994; doi:10.1371/journal.pgen.1003530)
Supplement: Table S2 — Clinical characteristics of the Danish samples. Data are mean ± standard deviation or median (interquartile range). (PDF) [file pgen.1003530.s004.pdf]

| **Table S2**. Clinical characteristics of the Danish samples | | |
|---|---|---|
| | Danish – Inter99 | Danish – Health2006 |
| N ($B_{12}$) | 5,481 | 2,812 |
| N (folate) | 5,624 | 2,804 |
| Men (%) | 49.2 | 45.1% |
| Age (yrs) | 46.2 ± 7.9 | 49.2 ± 13.1 |
| $B_{12}$ (pmol/l) | 280 (215-378) | 369 (295-243) |
| Folate (nmol/l) | 8.5 (6.2-12.3) | 15.9 (11.3-24.9) |

Data are mean ± standard deviation or median (interquartile range).
