# Supplementary material for: Genetic Architecture of Vitamin B12 and Folate Levels Uncovered Applying Deeply Sequenced Large Datasets
Source: PLoS Genet. 2013 Jun 6;9(6):e1003530. doi: 10.1371/journal.pgen.1003530 (PMC3674994; doi:10.1371/journal.pgen.1003530)
Supplement: Table S3 — Overview of the most significantly associated SNV for each of the identified B12 or folate loci in the Icelandic data. For each of the identified B12 or folate loci presented in Tables 1 and 2 the Icelandic association data for the lead SNV is shown. Moreover, the strongest associations at these loci in the Icelandic data are shown. The lead SNVs presented in Tables 1 and 2 are either the strongest signal at each of the loci or highly correlated with the strongest signal except at the FUT6 locus were rs708686 located 5′ of FUT6 gives the strongest signal. (PDF) [file pgen.1003530.s005.pdf]

**Table S3.** Overview of the most significantly associated SNV for each of the identified B_{12} or folate loci in the Icelandic data						
Locus	Chromosome	Locus lead SNV (Table 1 & 2)	Effect / *P* for locus lead SNV	Best Icelandic SNV in locus	Effect / *P* for best Icelandic SNV (if different from lead)	LD (r^2)
B_{12} associated loci						
CD320	19	rs2336573	0.32 / 1.1×10^{-51}	rs8109720	0.32 / 5.8×10^{-52}	0.97
TCN2	22	rs1131603	0.19 / 4.3×10^{-28}	rs1131603	-	-
ABCD4	14	rs3742801	0.045 / 5.3×10^{-8}	rs4619337	0.045 / 3.4×10^{-8}	1
MMAA	4	rs2270655	0.066 / 3.5×10^{-5}	rs114699496	-0.071 / 7.6×10^{-6}	0.94
MMACHC	1	rs12272669	0.51 / 3.0×10^{-9}	rs12272669	-	-
TCN1	11	rs34324219	0.21 / 8.8×10^{-71}	rs34324219	-	-
FUT6	19	rs778805	0.046 / 2.1×10^{-7}	rs708686	0.053 / 2.9×10^{-9}	0.64
FUT2	19	rs602662	0.16 / 4.1×10^{-96}	rs516316	0.17 / 3.6×10^{-103}	0.83
CUBN	10	rs1801222	0.11 / 1.1×10^{-52}	rs1801222	-	-
CLYBL	13	rs41281112	0.17 / 9.6×10^{-27}	rs41281112	-	-
MUT	6	rs1141321	0.061 / 1.4×10^{-16}	chr6:49508102	0.065 / 1.6×10^{-18}	0.95
Folate associated loci						
FOLR3	11	rs652197	0.069 / 2.5×10^{-10}	rs652197	-	-
MTHFR	1	rs1801133	0.096 / 1.0×10^{-28}	rs1801133	-	-

For each of the identified B_{12} or folate loci presented in Tables 1 and 2 the Icelandic association data for the lead SNV is shown. Moreover, the strongest associations at these loci in the Icelandic data are shown. The lead SNVs presented in Tables 1 and 2 are either the strongest signal at each of the loci or highly correlated with the strongest signal except at the *FUT6* locus were rs708686 located 5' of *FUT6* gives the strongest signal.
