# Supplementary material for: Genetic Architecture of Vitamin B12 and Folate Levels Uncovered Applying Deeply Sequenced Large Datasets
Source: PLoS Genet. 2013 Jun 6;9(6):e1003530. doi: 10.1371/journal.pgen.1003530 (PMC3674994; doi:10.1371/journal.pgen.1003530)
Supplement: Table S4 — Association results in the Icelandic data for SNVs previously reported to associate with B12 levels in GWAS. *These markers are only present in East-Asia. References: 1. Lin X, Lu D, Gao Y, Tao S, Yang X, et al. (2012) Genome-wide association study identifies novel loci associated with serum level of vitamin B12 in Chinese men. Hum Mol Genet 21: 2610–2617. 2. Hazra A, Kraft P, Lazarus R, Chen C, Chanock SJ, et al. (2009) Genome-wide significant predictors of metabolites in the one-carbon metabolism pathway. Hum Mol Genet 18: 4677–4687. 3. Hazra A, Kraft P, Selhub J, Giovannucci EL, Thomas G, et al. (2008) Common variants of FUT2 are associated with plasma vitamin B12 levels. Nat Genet 40: 1160–1162. (PDF) [file pgen.1003530.s006.pdf]

**Table S4.** Association results in the Icelandic data for SNVs previously reported to associate with B_{12} levels in GWAS								
**SNV name**	**Nearest gene**	**Chr.**	**Position (build 36/hg18)**	**Reference**	**Alleles (effect/ other)**	**EAF**	**Effect**	***P***
rs10515552*	None	5	145019024	[1]				
rs9473558	*MUT*	6	49520392	[2]	T/C	0.401	-0.061	1.4×10^{-16}
rs9473555	*MUT*	6	49517446	[2]	C/G	0.402	-0.062	5.4×10^{-17}
rs526934	*TCN1*	11	59390069	[2]	G/A	0.296	-0.119	2.3×10^{-48}
rs2298585	*MSRA*	11	59593768	[1]	T/C	0.001	0.214	0.075
rs601338	*FUT2*	19	53898486	[2,3]	G/A	0.384	-0.162	2.4×10^{-95}
rs1047781*	*FUT2*	19	53898443	[1]				
rs3760776	*FUT6*	19	5790746	[1]	A/G	0.071	0.068	4.4×10^{-6}

*These markers are only present in East-Asia. References: 1. Lin X, Lu D, Gao Y, Tao S, Yang X, et al. (2012) Genome-wide association study identifies novel loci associated with serum level of vitamin B12 in Chinese men. Hum Mol Genet 21: 2610-2617. 2. Hazra A, Kraft P, Lazarus R, Chen C, Chanock SJ, et al. (2009) Genome-wide significant predictors of metabolites in the one-carbon metabolism pathway. Hum Mol Genet 18: 4677-4687. 3. Hazra A, Kraft P, Selhub J, Giovannucci EL, Thomas G, et al. (2008) Common variants of *FUT2* are associated with plasma vitamin B12 levels. Nat Genet 40: 1160-1162.
