# Supplementary material for: Genetic Architecture of Vitamin B12 and Folate Levels Uncovered Applying Deeply Sequenced Large Datasets
Source: PLoS Genet. 2013 Jun 6;9(6):e1003530. doi: 10.1371/journal.pgen.1003530 (PMC3674994; doi:10.1371/journal.pgen.1003530)
Supplement: Table S5 — Association results in Icelandic data for SNVs previously reported with suggestive association with folate levels in GWAS. * Not previously shown at genome-wide significance. References: 1. Tanaka T, Scheet P, Giusti B, Bandinelli S, Piras MG, et al. (2009) Genome-wide association study of vitamin B6, vitamin B12, folate, and homocysteine blood concentrations. Am J Hum Genet 84: 477–482. 2. Hazra A, Kraft P, Lazarus R, Chen C, Chanock SJ, et al. (2009) Genome-wide significant predictors of metabolites in the one-carbon metabolism pathway. Hum Mol Genet 18: 4677–4687. (PDF) [file pgen.1003530.s007.pdf]

**Table S5.** Association results in Icelandic data for SNVs previously reported with suggestive association with folate levels in GWAS								
**SNV name**	**Nearest gene**	**Chr.**	**Position (build 36/hg18)**	**Reference**	**Alleles (effect/ other)**	**EAF**	**Effect**	***P***
rs1999594*	*MTHFR*	1	11881803	[1]	A/G	0.445	0.076	7.5×10^{-20}
rs982393*	*FIGN*	2	164198775	[2]	A/T	0.095	-0.017	0.24
rs2119289*	*FIGN*	2	164206192	[2]	C/G	0.095	-0.017	0.23
rs153734*	*PRICKLE2*	3	64053049	[1]	C/T	0.243	-0.003	0.71

***** Not previously shown at genome-wide significance. References: 1. Tanaka T, Scheet P, Giusti B, Bandinelli S, Piras MG, et al. (2009) Genome-wide association study of vitamin B6, vitamin B12, folate, and homocysteine blood concentrations. Am J Hum Genet 84: 477-482. 2. Hazra A, Kraft P, Lazarus R, Chen C, Chanock SJ, et al. (2009) Genome-wide significant predictors of metabolites in the one-carbon metabolism pathway. Hum Mol Genet 18: 4677-4687.
