# Supplementary material for: Genetic Architecture of Vitamin B12 and Folate Levels Uncovered Applying Deeply Sequenced Large Datasets
Source: PLoS Genet. 2013 Jun 6;9(6):e1003530. doi: 10.1371/journal.pgen.1003530 (PMC3674994; doi:10.1371/journal.pgen.1003530)
Supplement: Table S6 — Suggestive loci in the Icelandic or the Icelandic and Danish data associated with serum B12 levels (2.2×10^−9^> P<10−6). (PDF) [file pgen.1003530.s008.pdf]

Table S6. Suggestive loci in the Icelandic or the Icelandic and Danish data associated with serum B_{12} levels ($2.2 \times 10^{-9} > P < 10^{-6}$)

**SNV name**	**Locus/ nearest gene**	**Chr.**	**Position (build 36/hg18)**	**Annotation**	**Alleles (effect / other)**	**EAF**	**Icelandic**		**Danish – Inter99**		**Danish – Health2006**		**Combined**		
							**Effect**	***P***	**Effect**	***P***	**Effect**	***P***	**N**	***P***	I^2 (P_{HET})
rs1047891	*CPS1*	2	211,248,752	T1406N	**C**/A	0.628	0.038	7.6×10^{-6}	0.097	5.5×10^{-4}	0.034	0.11	45,574	3.0×10^{-8}	0 (0.15)
Chr6_88792234	*SPACA1*	6	88792234	Intergenic	G/A	0.0058	0.262	2.8×10^{-7}	NA	NA	NA	NA	NA	NA	NA
rs62515066	*ZBTB10*	8	81510638	Intergenic	G/A	0.0252	0.121	5.4×10^{-7}	NA	NA	NA	NA	NA	NA	NA
