# Supplementary material for: Genetic Architecture of Vitamin B12 and Folate Levels Uncovered Applying Deeply Sequenced Large Datasets
Source: PLoS Genet. 2013 Jun 6;9(6):e1003530. doi: 10.1371/journal.pgen.1003530 (PMC3674994; doi:10.1371/journal.pgen.1003530)
Supplement: Table S7 — Results in the Icelandic data for loci with suggestive association with serum folate levels (2.2×10^−9^>P<10−6). (PDF) [file pgen.1003530.s009.pdf]

Table S7. Results in the Icelandic data for loci with suggestive association with serum folate levels ($2.2 \times 10^{-9} > P < 10^{-6}$)

SNV name	Locus	Chr.	Position (build 36/hg18)	Alleles (effect/ other)	EAF	Icelandic	
						**Effect**	**P**
rs71466511	*CCDC34*	11	27,277,877	G/**A**	0.480	0.048	4.7×10^{-8}
chr14:103981131	*TMEM179*	14	103,981,131	T/C	0.0032	-0.50	2.7×10^{-7}
chr15:45048726	*MIR548A3*	15	45,048,726	T/G	0.018	0.18	4.8×10^{-7}
chr17:8017826	*TMEM107*	17	8,017,826	C/T	0.0022	-0.51	4.9×10^{-7}
chr13:95871811	*HS6ST3*	13	95,871,811	G/T	0.0052	0.32	5.2×10^{-7}
chr3:12140862	*SYN2*	3	12,140,862	T/G	0.0115	0.19	$8,8 \times 10^{-7}$
chr12:114065998	*TBX3*	12	114,065,998	A/C	0.0206	-0.15	8.8×10^{-7}
rs61675507	*PCDHGA6*	5	140,741,961	T/G	0.2035	-0.051	9.2×10^{-7}
