# Supplementary material for: Genetic Architecture of Vitamin B12 and Folate Levels Uncovered Applying Deeply Sequenced Large Datasets
Source: PLoS Genet. 2013 Jun 6;9(6):e1003530. doi: 10.1371/journal.pgen.1003530 (PMC3674994; doi:10.1371/journal.pgen.1003530)
Supplement: Table S8 — Results from stepwise conditional analyses using the Icelandic data at loci associated with serum B12 or folate levels for signals with P<5×10^−8^. Conditional analyses were performed using imputed sequence data from chip-genotyped Icelanders with information on serum B12 or folate levels. Results for SNV #1 (lead SNVs) at each loci are unconditional on other SNVs. Analysis of SNV #2 is conditional on SNV #1 and SNV #3 is conditional on SNV #1 and #2. The LD between the SNVs at each locus was estimated from the sequence information of the 1,179 whole genome sequenced Icelanders. (PDF) [file pgen.1003530.s010.pdf]

Table S8. Results from stepwise conditional analyses using the Icelandic data at loci associated with serum B_{12} or folate levels for signals with $P < 5 \times 10^{-8}$

SNV #	SNV name	Chr.	Position (build 36)	Gene	Annotation	Alleles (effect/ other)	EAF	Unconditional		Conditional on SNV #1		Conditional on SNVs #1 and #2		LD with SNV #1	LD with SNV #2
								Effect	*P*	Effect	*P*	Effect	*P*	r^2	r^2
***CUBN* region**															
1	rs1801222	10	17,196,157	*CUBN*	F253S	G/A	0.593	0.11	2.3×10^{-42}						
2	rs56077122	10	17,247,021	*TRDMT1*	intronic	A/C	0.335	0.061	1.6×10^{-9}	0.0866	4.8×10^{-21}			0.033	
***TCN1* region**															
1	rs34324219	11	59,379,954	*TCN1*	D301Y	C/A	0.889	0.21	9.8×10^{-62}						
2	rs34528912	11	59,388,111	*TCN1*	R35H	T/C	0.0361	0.15	2.5×10^{-12}	0.17	2.1×10^{-15}			0.0040	
3	rs117456053	11	59,373,407	Near *TCN1*	Intergenic	G/A	0.976	0.12	4.1×10^{-6}	0.15	1.2×10^{-8}	0.16	1.9×10^{-9}	0.0035	0.0011
***TCN2* region**															
1	rs1131603	22	29,348,975	*TCN2*	L376S	C/T	0.055	0.17	1.1×10^{-21}						
2	rs5753231	22	29,333,069	*TCN2*	Promoter	C/T	0.79	0.053	1.9×10^{-7}	0.064	7.5×10^{-10}			0.014	
***MTHFR* region**															
1	rs1801133	1	11,778,965	*MTHFR*	A222V	G/A	0.668	0.10	3.4×10^{-27}						
2	rs17421511	1	11,780,375	*MTHFR*	Intronic	G/A	0.827	0.045	0.00011	0.098	1.8×10^{-15}			0.11	

Conditional analyses were performed using imputed sequence data from chip-typed Icelanders with information on serum B_{12} or folate levels. Results for SNV #1 (lead SNVs) at each loci are unconditional on other SNVs. Analysis of SNV #2 is conditional on SNV #1 and SNV #3 is conditional on SNV #1 and #2. The LD between the SNVs at each locus was estimated from the sequence information of the 1,179 whole genome sequenced Icelanders.
