# Supplementary material for: Genetic Architecture of Vitamin B12 and Folate Levels Uncovered Applying Deeply Sequenced Large Datasets
Source: PLoS Genet. 2013 Jun 6;9(6):e1003530. doi: 10.1371/journal.pgen.1003530 (PMC3674994; doi:10.1371/journal.pgen.1003530)
Supplement: Table S9 — Cis -effect of the B12 and folate SNVs on the expression of the target gene in white blood cells and adipose tissue. Correlation between SNVs that associate with increased B12 or folate and mRNA expression in blood and adipose tissue from 1,001 and 673 individuals, respectively. The correlations are tested by regression analysis adjusted, for age, sex and differential cell counts (blood only), and inverse normal transformed relative expression values on the estimated genotype dosage. aNo other SNV shows significantly higher correlation with the expression in adipose or blood of MUT than rs1141321. bThe INDEL chr11:71527804 is the most significant cis variant for FOLR3. cFor TCN2 there are cis variants both in blood and adipose tissue that have stronger correlation than rs5753231 with its expression, while having little effect on B12 levels. (PDF) [file pgen.1003530.s011.pdf]

Table S9. *Cis*-effect of the B_{12} and folate SNVs on the expression of the target gene in white blood cells and adipose tissue.

								**Blood**		**Adipose**	
**SNV name**	**ID**	**Trait**	**Alleles (effect/other)**	**EAF**	**Trait effect**	**Gene**	**Transcript**	**Effect**	***P***	**Effect**	***P***
rs1801133	chr1:11778965	Folate	G/A	0.653	0.096	*MTHFR*	NM_005957	0.027	0.59	-0.039	0.53
rs17421511	chr1:11780375	Folate	G/A	0.829	0.044	*MTHFR*	NM_005957	0.126	0.035	0.141	0.054
rs12272669	chr1:45747242	B_{12}	A/G	0.0028	0.363	*MMACHC*	AL080062	0.358	0.49	-0.101	0.85
rs2270655	chr4:146795868	B_{12}	G/C	0.946	0.081	*MMAA*	NM_172250	0.137	0.17	-0.323	0.012
rs1141321[a]	chr6:49520392	B_{12}	C/T	0.599	0.065	*MUT*	NM_000255	-0.736	9.1×10^{-59}	-0.483	2.5×10^{-16}
rs1801222	chr10:17196157	B_{12}	G/A	0.581	0.112	*CUBN*	NM_001081	-0.052	0.27	-0.094	0.1
rs56077122	chr10:17247021	B_{12}	A/C	0.333	0.062	*CUBN*	NM_001081	0.096	0.057	0.084	0.17
rs34324219	chr11:59379954	B_{12}	C/A	0.891	0.218	*TCN1*	NM_001062	0.180	0.021	-0.044	0.63
rs34528912	chr11:59388111	B_{12}	C/T	0.963	0.153	*TCN1*	NM_001062	0.263	0.022	-0.376	0.018
rs117456053	chr11:59373407	B_{12}	G/A	0.976	0.126	*TCN1*	NM_001062	-0.079	0.57	0.073	0.69
rs652197[b]	chr11:71527389	Folate	C/T	0.178	0.067	*FOLR3*	NM_000804	0.997	2.6×10^{-69}	0.896	8.0×10^{-37}
rs139130389 (INDEL)[b]	chr11:71527804	Folate	CTA/C	0.101	0.087	*FOLR3*	NM_000804	1.514	7.1×10^{-110}	1.367	1.8×10^{-62}
rs41281112	chr13:99316635	B_{12}	C/T	0.946	0.164	*CLYBL*	NM_138280	0.151	0.14	0.051	0.71
rs3742801	chr14:73828759	B_{12}	T/C	0.273	0.041	*ABCD4*	NM_005050	0.069	0.17	-0.058	0.35
rs3742801	chr14:73828759	B_{12}	T/C	0.273	0.041	*ABCD4*	NM_020323	0.116	0.018	0.098	0.11
rs778805	chr19:5783209	B_{12}	A/G	0.237	0.044	*FUT6*	NM_000150	-0.145	0.0084	-0.097	0.15
rs708686	chr19:5791619	B_{12}	T/C	0.293	0.055	*FUT6*	NM_000150	-0.160	0.0037	-0.037	0.58
rs602662	chr19:53898797	B_{12}	A/G	0.633	0.166	*FUT2*	NM_000511	0.004	0.93	-0.021	0.73
rs2336573	chr19:8273709	B_{12}	T/C	0.330	0.318	*CD320*	NM_016579	-0.756	2.6×10^{-07}	0.482	0.024
rs1131603	chr22:29348975	B_{12}	C/T	0.519	0.195	*TCN2*	NM_000355	0.310	0.0028	0.406	0.0027
rs5753231[c]	chr22:29333069	B_{12}	T/C	0.208	0.047	*TCN2*	NM_000355	-0.266	6.5×10^{-06}	-0.440	7.0×10^{-10}

Correlation between SNVs that associate with increased B_{12} or folate and mRNA expression in blood and adipose tissue from 1,001 and 673 individuals, respectively. The correlations are tested by regression analysis adjusted, for age, sex and differential cell counts (blood only), and inverse normal transformed relative expression values on the estimated genotype dosage.

[a]No other SNV shows significantly higher correlation with the expression in adipose or blood of *MUT* than rs1141321.

[b]The INDEL chr11:71527804 is the most significant *cis* variant for *FOLR3.*

[c]For *TCN2* there are *cis*-variants both in blood and adipose tissue that have stronger correlation than rs5753231with its expression, while having little effect on serum B_{12} levels.
