# Supplementary material for: Genetic Architecture of Vitamin B12 and Folate Levels Uncovered Applying Deeply Sequenced Large Datasets
Source: PLoS Genet. 2013 Jun 6;9(6):e1003530. doi: 10.1371/journal.pgen.1003530 (PMC3674994; doi:10.1371/journal.pgen.1003530)
Supplement: Table S10 — Association results for B12 and folate associated markers with potential co-morbid conditions in Icelanders. a Effect size and effect allele frequency from the Icelandic population. Associations at P<0.001 are shown in bold. EAF, effect allele frequency; MCV, mean corpuscular volume; MCH, mean corpuscular hemoglobin. (PDF) [file pgen.1003530.s012.pdf]

Table S10. Association results for B_{12} and folate associated markers with potential co-morbid conditions in Icelanders

		Discovery B_{12} or folate		Coronary artery disease (21,507 cases 86.028 contr.)		Stroke (3,829 cases 107,212 contr.)		Colorectal Cancer (3,450 cases 69,000 contr.)		Prostate Cancer (4,792 cases 71,880 contr.)		Alzheimer (3,550 cases 110,050 controls)		MCV (49,385)		MCH (49,385)		Creatinine (44,397)		Homocysteine (2,297)	
	**Gene**	**Effect**	**EAF**	***P***	**OR**	***P***	**OR**	***P***	**OR**	***P***	**OR**	***P***	**OR**	**Effect**	***P***	**Effect**	***P***	**Effect**	***P***	**Effect**	***P***
B_{12} associated SNVs																					
rs12272669	*MMACHC*	0.51	0.0026	0.051	0.739	0.42	0.788	0.91	1.042	0.42	0.783	0.53	0.800	-0.042	0.46	-0.032	0.56	-0.04	0.41	-0.13	0.67
rs2336573	*CD320*	0.32	0.033	0.07	0.923	0.69	0.969	0.54	1.068	0.15	1.120	0.20	1.127	-0.011	0.51	0	0.97	-0.003	0.82	0.022	0.73
rs34324219	*TCN1*	0.21	0.891	0.79	1.007	0.83	1.010	0.10	1.116	0.023	1.114	0.92	0.994	0.003	0.76	0.001	0.88	0.002	0.82	0.03	0.4
rs1131603	*TCN2*	0.19	0.051	0.55	0.979	0.25	1.074	0.13	1.140	0.30	1.068	0.28	1.087	0.023	0.08	0.021	0.09	0.005	0.64	0.016	0.76
rs41281112	*CLYBL*	0.17	0.944	0.092	0.945	0.05	0.887	0.71	0.967	0.39	1.057	0.65	1.035	-0.017	0.16	-0.013	0.28	-0.0008	0.90	-0.043	0.39
rs34528912	*TCN1*	0.17	0.035	0.81	1.010	0.63	0.964	0.61	0.947	0.65	0.965	0.97	0.996	-0.003	0.83	0.003	0.85	0.008	0.53	0.034	0.56
rs602662	*FUT2*	0.16	0.634	0.75	1.005	0.79	1.008	0.19	1.057	0.58	0.983	0.21	1.047	0.004	0.54	0.004	0.51	0.013	0.007	-0.012	0.61
rs117456053	Near *TCN1*	0.16	0.976	0.55	0.970	0.11	1.168	0.80	1.035	0.32	0.912	0.29	1.126	0.019	0.33	0.023	0.22	-0.01	0.37	-0.124	0.08
rs1801222	*CUBN*	0.11	0.582	**0.00085**	**1.053**	0.058	1.056	0.59	0.978	0.18	1.041	0.22	1.045	0.004	0.48	0.004	0.52	0.007	0.12	-0.025	0.29
rs56077122	*CUBN*	0.087	0.333	0.54	0.990	0.58	0.983	0.66	1.018	0.44	0.977	0.29	0.962	-0.007	0.27	-0.004	0.49	0.007	0.13	0.007	0.77
rs2270655	*MMAA*	0.066	0.949	0.82	1.008	0.74	0.979	0.54	1.057	0.14	0.911	0.57	0.957	0.008	0.53	0.005	0.68	-0.03	0.002	0.022	0.67
rs5753231	*TCN2*	0.064	0.793	0.15	0.974	0.59	0.981	0.62	0.976	0.10	0.943	0.71	1.016	0.003	0.63	0.007	0.34	-0.002	0.71	-0.063	0.03
rs1141321	*MUT*	0.061	0.6	0.02	0.965	0.79	1.008	0.96	1.002	0.73	0.990	0.50	0.976	-0.001	0.85	-0.003	0.5	3×10^{-04}	0.94	**-0.07**	**0.001**
rs708686	*FUT6*	0.046	0.235	0.98	1.000	0.11	1.055	0.34	1.046	0.72	0.988	1.00	1.000	0.005	0.44	0.004	0.59	1×10^{-04}	0.93	0.01	0.72
rs3742801	*ABCD4*	0.045	0.275	0.41	0.986	0.48	0.978	0.42	1.036	0.21	1.041	0.36	1.036	0.009	0.14	0.01	0.13	0.002	0.7	-0.008	0.76
Folate associated SNVs																					
rs17421511	*MTHFR*	0.098	0.83	0.41	0.984	**0.00086**	**0.885**	0.78	0.985	0.94	0.997	0.61	0.977	-0.007	0.37	-0.004	0.55	-0002	0.82	-0.093	0.002
rs1801133	*MTHFR*	0.096	0.655	0.31	1.016	0.62	1.015	0.026	1.099	0.45	1.023	0.82	1.008	-0.013	0.03	-0.016	0.007	-0.01	0.08	**-0.094**	**9.8×10^{-5}**
rs139130389 (INDEL)	*FOLR3*	0.087	0.104	0.65	0.989	0.83	1.010	0.91	0.992	0.04	1.104	0.24	0.933	0.002	0.82	-0.001	0.87	-0.01	0.42	0.059	0.11
rs652197	*FOLR3*	0.069	0.178	0.43	1.016	0.73	0.987	0.59	1.028	0.018	1.092	0.22	0.945	0.002	0.75	0	0.98	0.003	0.64	0.029	0.34

[a] Effect size and effect allele frequency from the Icelandic population

Associations at $P < 0.001$ are shown in bold.

EAF, effect allele frequency; MCV, mean corpuscular volume; MCH, mean corpuscular hemoglobin.
