# Supplementary material for: Genetic Architecture of Vitamin B12 and Folate Levels Uncovered Applying Deeply Sequenced Large Datasets
Source: PLoS Genet. 2013 Jun 6;9(6):e1003530. doi: 10.1371/journal.pgen.1003530 (PMC3674994; doi:10.1371/journal.pgen.1003530)
Supplement: Table S11 — Association results for the B12 and folate variants with diseases and traits in deCODE database. Shown are the strongest association results for the folate and B12 variants, genome-wide significant or suggestive, with diseases and traits in deCODE's database. EAF, effect allele frequency; BMD, bone mineral density; AAA, abdominal aortic aneurysm; TA, thoracic aneurysm; 1The annotation is based on the RefSeq hg18, 2The reference alleles based on Build 36 hg18 are shown in bold, 3The low BMD phenotypes are defined as those BMD values that are below −1 standard deviation (SD) from the mean. (PDF) [file pgen.1003530.s013.pdf]

Table S11. Association results for the B_{12} and folate variants with diseases and traits in deCODE database

Condition	N cases/ controls	Annotation[1]	Allele[2] (effect/ other)	EAF	Effect	OR	*P*
FUT2 (rs60266) - multiplicative model							
B_{12}	37,283	G258S	A/**G**	0.596	0.16		4.1×10^{-96}
Cancer antigen 19 9	4,493	G258S	A/**G**	0.596	0.48		1.1×10^{-146}
Liver alkaline phosphatase	49,840	G258S	A/**G**	0.596	-0.08		1.1×10^{-73}
Lipase	14,929	G258S	A/**G**	0.596	0.10		2.2×10^{-24}
Psoriasis	1,657/92,792	G258S	A/**G**	0.596		1.14	0.0043
Low BMD[3]		G258S	A/**G**				
Hip, spine or whole body	4,931/69,034	G258S	A/**G**	0.596		1.10	4.6×10^{-4}
Spine	3,015/41,800	G258S	A/**G**	0.596		1.16	1.3×10^{-5}
FUT6 (7788053) - recessive model							
B_{12}	37,283	P124S	A/**G**	0.254	0.046		2.1×10^{-7}
AAA	1,163/61,639	P124S	A/**G**	0.254		0.65	0.037
AAA <65 years	209/41,800	P124S	A/**G**	0.254		0.21	0.010
MTHFR (rs18011339) - recessive model							
Folate	28,913	A222V	**G**/A	0.668	0.096		1.0×10^{-28}
TA	219/31,536	A222V	**G**/A	0.668		0.48	0.0056

Shown are the strongest association results for the folate and B_{12} variants, genome-wide significant or suggestive, with diseases and traits in deCODE's database.

EAF, effect allele frequency; BMD, bone mineral density; AAA, abdominal aortic aneurysm; TA, thoracic aneurysm;

[1]The annotation is based on the RefSeq hg18

[2]The reference alleles based on Build 36 hg18 are shown in bold

[3] The low BMD phenotypes are defined as those BMD values that are below -1 standard deviation (SD) from the mean.
