# Supplementary material for: Genetic Architecture of Vitamin B12 and Folate Levels Uncovered Applying Deeply Sequenced Large Datasets
Source: PLoS Genet. 2013 Jun 6;9(6):e1003530. doi: 10.1371/journal.pgen.1003530 (PMC3674994; doi:10.1371/journal.pgen.1003530)
Supplement: Text S1 — Supplementary Text S1 contains additional description of Methods. (PDF) [file pgen.1003530.s014.pdf]

SUPPLEMENTARY INFORMATION

Genetic architecture of vitamin B_{12} and folate levels uncovered applying deeply sequenced large datasets

Niels Grarup[1]*, Patrick Sulem[2]*, Camilla H. Sandholt[1]*, Gudmar Thorleifsson[2], Tarunveer S. Ahluwalia[1], Valgerdur Steinthorsdottir[2], Helgi Bjarnason[2], Daniel F. Gudbjartsson[2], Olafur T. Magnusson[2], Thomas Sparsø[1], Anders Albrechtsen[3], Augustine Kong[2], Gisli Masson[2], Geng Tian[4], Hongzhi Cao[4], Chao Nie[4], Karsten Kristiansen[5], Lise Lotte Husemoen[6], Betina Thuesen[6], Yingrui Li[4], Rasmus Nielsen[3,7,8], Allan Linneberg[6], Isleifur Olafsson[9], Gudmundur I. Eyjolfsson[10], Torben Jørgensen[6,11,12], Jun Wang[1,4,5], Torben Hansen[1,13], Unnur Thorsteinsdottir[2,14], Kari Stefánsson[2,14], Oluf Pedersen[1,15,16,17]

[1] The Novo Nordisk Foundation Center for Basic Metabolic Research, Faculty of Health and Medical Sciences, University of Copenhagen, Copenhagen, Denmark
[2] deCODE Genetics, Reykjavik, Iceland
[3] Centre of Bioinformatics, Faculty of Science, University of Copenhagen, Copenhagen, Denmark
[4] BGI-Shenzhen, Shenzhen, China
[5] Department of Biology, Faculty of Science, University of Copenhagen, Copenhagen, Denmark.
[6] Research Centre for Prevention and Health, Glostrup University Hospital, Glostrup, Denmark
[7] Department of Integrative Biology, University of California, Berkeley, CA, US
[8] Department of Statistics, University of California, Berkeley, CA, US
[9] Landspitali, The National University Hospital of Iceland, Department of Clinical Biochemistry, Reykjavik, Iceland
[10] Icelandic Medical Center (Laeknasetrid) Laboratory in Mjodd (RAM), Reykjavik, Iceland
[11] Faculty of Health and Medical Sciences, University of Copenhagen, Copenhagen, Denmark
[12] Faculty of Medicine, University of Aalborg, Aalborg, Denmark
[13] Faculty of Health Sciences, University of Southern Denmark, Odense, Denmark
[14] University of Iceland Faculty of Medicine, Reykjavik, Iceland
[15] Faculty of Health Sciences, Aarhus University, Aarhus, Denmark
[16] Hagedorn Research Institute, Gentofte, Denmark
[17] Institute of Biomedical Science, Faculty of Health and Medical Sciences, University of Copenhagen, Copenhagen, Denmark

Supplementary Text

Whole genome sequencing

Single nucleotide variants (SNVs) were identified through the Icelandic whole genomic sequencing project. A total of 1,176 Icelanders were selected for sequencing based on having various neoplasic, cardiovascular and psychiatric conditions. All of the individuals were sequenced to a depth of at least 10×.

Sample preparation. Paired-end libraries for sequencing were prepared according to the manufacturer's instructions (Illumina). In short, approximately 5 µg of genomic DNA, isolated from frozen blood samples, was fragmented to a mean target size of 300 bp using a Covaris E210 instrument. The resulting fragmented DNA was end repaired using T4 and Klenow polymerases and T4 polynucleotide kinase with 10 mM dNTP followed by addition of an 'A' base at the ends using Klenow exo fragment (3' to 5'-exo minus) and dATP (1 mM). Sequencing adaptors containing 'T' overhangs were ligated to the DNA products followed by agarose (2%) gel electrophoresis. Fragments of about 400 bp were isolated from the gels (QIAGEN Gel Extraction Kit), and the adaptor-modified DNA fragments were PCR enriched for ten cycles using Phusion DNA polymerase (Finnzymes Oy) and PCR primers PE 1.0 and PE 2.0 (Illumina). Enriched libraries were further purified using agarose (2%) gel electrophoresis as described above. The quality and concentration of the libraries were assessed with the Agilent 2100 Bioanalyzer using the DNA 1000 LabChip (Agilent). Barcoded libraries were stored at −20 °C. All steps in the workflow were monitored using an in-house laboratory information management system with barcode tracking of all samples and reagents.

DNA sequencing. Template DNA fragments were hybridized to the surface of flow cells (Illumina PE flowcell, v4) and amplified to form clusters using the Illumina cBot. In brief, DNA (8–10 pM) was denatured, followed by hybridization to grafted adaptors on the flowcell. Isothermal bridge amplification using Phusion polymerase was then followed by linearization of the bridged DNA, denaturation, blocking of 3 ends and hybridization of the sequencing primer. Sequencing-by-synthesis was performed on Illumina GAIIx instruments equipped with paired-end modules. Paired-end libraries were sequenced using 2 × 101 cycles of incorporation and imaging with Illumina sequencing kits, v4. Each library or sample was initially run on a single lane for validation followed by further sequencing of ≥4 lanes with targeted cluster densities of 250–300 k/mm^2. Imaging and analysis of the data was performed using the SCS 2.6 and RTA 1.6 software packages from Illumina, respectively. Real-time analysis involved conversion of image data to base-calling in real-time.

Alignment. For each lane in the DNA sequencing output, the resulting qseq files were converted into fastq files using an in-house script. All output from sequencing was converted, and the Illumina quality filtering

flag was retained in the output. The fastq files were then aligned against Build 36 of the human reference sequence using version 0.5.7 of bwa [1].

BAM file generation. SAM file output from the alignment was converted into BAM format using version 0.1.8 of samtools [2], and an in-house script was used to carry the Illumina quality filter flag over to the BAM file. The BAM files for each sample were then merged into a single BAM file using samtools. Finally, Picard version 1.17 (see http://picard.sourceforge.net/) was used to mark duplicates in the resulting sample BAM files.

SNV calling and genotyping in whole genome sequencing

A two-step approach was applied. The first step was to detect SNVs by identifying sequence positions where at least one individual could be determined to be different from the reference sequence with confidence (quality threshold of 20) based on the SNV calling feature of the pileup tool samtools [2]. SNVs that always differed heterozygous or homozygous from the reference were removed. The second step was to use the pileup tool to genotype the SNVs at the positions that were flagged as polymorphic. Because sequencing depth varied and hence the certainty of genotype calls also varied, genotype likelihoods rather than deterministic calls were calculated (see below). Of the 2.5 million SNVs reported in the HapMap2 CEU samples, 96.3% were observed in the whole genome sequencing data. Of the 6.9 million SNVs reported in the 1000 Genomes Project data, 89.4% were observed in the whole genome sequencing data.

Long range phasing

Long range phasing of all chip-genotyped individuals was performed with methods described previously [3,4]. In brief, phasing is achieved using an iterative algorithm which phases a single proband at a time given the available phasing information about everyone else that shares a long haplotype identically by state with the proband. Given the large fraction of the Icelandic population that has been chip-typed, accurate long range phasing is available genome-wide for all chip-typed Icelanders.

Genotype imputation

We imputed the SNVs identified and genotyped through sequencing into all Icelanders who had been phased with long range phasing using the same model as used by IMPUTE [5]. The genotype data from sequencing can be ambiguous due to low sequencing coverage. In order to phase the sequencing genotypes, an iterative algorithm was applied for each SNV with alleles 0 and 1. We let H be the long range phased haplotypes of the sequenced individuals and applied the following algorithm:

1. For each haplotype h in H, use the Hidden Markov Model of IMPUTE to calculate for every other k in H, the likelihood, denoted $\gamma_{h,k}$, of h having the same ancestral source as k at the SNV.
2. For every h in H, initialize the parameter θ_h, which specifies how likely the one allele of the SNV is to occur on the background of h from the genotype likelihoods obtained from sequencing. The genotype likelihood L_g is the probability of the observed sequencing data at the SNV for a given individual assuming g is the true genotype at the SNV. If L_0, L_1 and L_2 are the likelihoods of the genotypes 0, 1 and 2 in the individual that carries h, then set $\theta_h = \frac{L_2 + \frac{1}{2}L_1}{L_2 + L_1 + L_0}$.
3. For every pair of haplotypes h and k in H that are carried by the same individual, use the other haplotypes in H to predict the genotype of the SNV on the backgrounds of h and k: $\tau_h = \sum_{l \in H \setminus \{h\}} \gamma_{h,l}\theta_l$ and $\tau_k = \sum_{l \in H \setminus \{k\}} \gamma_{k,l}\theta_l$. Combining these predictions with the genotype likelihoods from sequencing gives un-normalized updated phased genotype probabilities: $P_{00} = (1 - \tau_h)(1 - \tau_k)L_0$, $P_{10} = \tau_h(1 - \tau_k)\frac{1}{2}L_1$, $P_{01} = (1 - \tau_h)\tau_k\frac{1}{2}L_1$ and $P_{11} = \tau_h\tau_k L_2$. Now use these values to update ϑ_h and ϑ_k to $\theta_h = \frac{P_{10} + P_{11}}{P_{00} + P_{01} + P_{10} + P_{11}}$ and $\theta_k = \frac{P_{01} + P_{11}}{P_{00} + P_{01} + P_{10} + P_{11}}$.
4. Repeat step 3 when the maximum difference between iterations is greater than a convergence threshold ε. We used $\varepsilon = 10^{-7}$.

Given the long range phased haplotypes and θ_l the allele of the SNV on a new haplotype h not in H, is imputed as $\sum_{l \in H} \gamma_{h,l}\theta_l$.

The above algorithm can easily be extended to handle simple family structures such as parent-offspring pairs and triads by letting the P distribution run over all founder haplotypes in the family structure. The algorithm also extends trivially to the X-chromosome. If source genotype data are only ambiguous in phase, such as chip-genotype data, then the algorithm is still applied, but all but one of the Ls will be 0. In some instances, the reference set was intentionally enriched for carriers of the minor allele of a rare SNV in order to improve imputation accuracy. In this case, expected allele counts will be biased toward the minor allele of the SNV. Call the enrichment of the minor allele E and let θ' be the expected minor allele count calculated from the naïve imputation method, and let θ be the unbiased expected allele count, then $\theta' = \frac{E\theta}{1 - \theta + E\theta}$ and hence $\theta = \frac{\theta'}{E + (1 - E)\theta'}$.

This adjustment was applied to all imputations based on enriched imputations sets. We note that if θ' is 0 or 1, then θ will also be 0 or 1, respectively.

Genotype imputation information

The informativeness of genotype imputation was estimated by the ratio of the variance of imputed expected allele counts and the variance of the actual allele counts:

$$\frac{Var(E(\theta|chip\ data))}{Var(\theta)},$$

where $\theta \in \{0, 1\}$ is the allele count. $Var(E(\theta|chip\ data))$ was estimated by the observed variance of the imputed expected counts and $Var(\theta)$ was estimated by $p(1-p)$, where p is the allele frequency.

***In silico* genotyping**

In addition to imputing sequence variants from the whole genome sequencing effort into chip-genotyped individuals, we also performed a second imputation step where genotypes were imputed into relatives of chip-genotyped individuals, creating *in silico* genotypes. The inputs into the second imputation step are the fully phased (in particular every allele has been assigned a parent of origin) imputed and chip-type genotypes of the available chip-typed individuals. The algorithm used to perform the second imputation step consists of:

1. For each ungenotyped individual (the proband), find all chip-genotyped individuals within two meiosis of the individual. The six possible types of two meiosis relatives of the proband are (ignoring more complicated relationships due to pedigree loops): Parents, full and half siblings, grandparents, children and grandchildren. If all pedigree paths from the proband to a genotyped relative go through other genotyped relatives, then that relative is excluded. E.g. if a parent of the proband is genotyped, then the proband's grandparents through that parent are excluded. If the number of meiosis in the pedigree around the proband exceeds a threshold (we used 12), then relatives are removed from the pedigree until the number of meiosis falls below 12, in order to reduce computational complexity.
2. At every point in the genome, calculate the probability for each genotyped relative sharing with the proband based on the autosomal SNVs used for phasing. A multipoint algorithm based on the Hidden Markov model Lander-Green multipoint linkage algorithm using fast Fourier transforms is used to calculate these sharing probabilities [6,7]. First single point sharing probabilities are calculated by dividing the genome into 0.5 cM bins and using the haplotypes over these bins as alleles. Haplotypes that are the same, except at most at a single SNV, are treated as identical. When the haplotypes in the pedigree are incompatible over a bin, then a uniform probability distribution was used for that bin. The most common causes for such incompatibilities are recombinations

within the pedigree, phasing errors and genotyping errors. Note that since the input genotypes are fully phased, the single point information is substantially more informative than for unphased genotyped, in particular one haplotype of the parent of a genotyped child is always known. The single point distributions are then convolved using the multipoint algorithm to obtain multipoint sharing probabilities at the center of each bin. Genetic distances were obtained from the most recent version of the deCODE genetic map[4].

3. Based on the sharing probabilities at the center of each bin, all the SNVs from the whole genome sequencing are imputed into the proband. To impute the genotype of the paternal allele of a SNV located at x, flanked by bins with centers at x_{left} and x_{right}. Starting with the left bin, going through all possible sharing patterns v, let I_v be the set of haplotypes of genotyped individuals that share identically by descent within the pedigree with the proband's paternal haplotype given the sharing pattern v and $P(v)$ be the probability of v at the left bin – this is the output from step 2 above – and let e_i be the expected allele count of the SNV for haplotype i. Then $e_v = \frac{\sum_{i \in I_v} e_i}{\sum_{i \in I_v} 1}$ is the expected allele count of the paternal haplotype of the proband given v and an overall estimate of the allele count given the sharing distribution at the left bin is obtained from $e_{left} = \sum_v P(v) e_v$. If I_v is empty then no relative shares with the proband's paternal haplotype given v and thus there is no information about the allele count. We therefore store the probability that some genotyped relative shared the proband's paternal haplotype, $O_{left} = \sum_{v, I_v = \emptyset} P(V)$ and an expected allele count, conditional on the proband's paternal haplotype being shared by at least one genotyped relative: $c_{left} = \frac{\sum_{v, I_v \neq \emptyset} P(v) e_v}{\sum_{v, I_v \neq \emptyset} P(v)}$. In the same way calculate O_{right} and c_{right}. Linear interpolation is then used to get an estimates at the SNV from the two flanking bins:

$$O = O_{left} + \frac{x - x_{left}}{x_{right} - x_{left}} \left(O_{right} - O_{left} \right),$$

$$c = c_{left} + \frac{x - x_{left}}{x_{right} - x_{left}} \left(c_{right} - c_{left} \right).$$

If θ is an estimate of the population frequency of the SNV then $Oc + (1 - O)\theta$ is an estimate of the allele count for the proband's paternal haplotype. Similarly, an expected allele count can be obtained for the proband's maternal haplotype.

Quantitative trait association testing

A generalized form of linear regression was used to test for association of serum vitamin B_{12} (B_{12}) and folate with SNVs. Let y be the vector of quantitative measurements, and let g be the vector of expected allele

counts for the SNV being tested. We assume the quantitative measurements follow a normal distribution with a mean that depends linearly on the expected allele at the SNV and a variance covariance matrix proportional to the kinship matrix:

$$y \sim \mathcal{N}(\alpha + \beta g, 2\sigma^2 \Phi),$$

where

$$\Phi_{ij} = \begin{cases} \frac{1}{2}, i = j \\ 2\rho k_{ij}, i \neq j \end{cases}.$$

is based on the kinship between individuals as estimated from the Icelandic genealogical database (k_{ij}) and and estimate of the heritability of the trait (ρ). It is not computationally feasible to use this full model and we therefore split the individuals with *in silico* genotypes and B_{12} and folate measurements into smaller clusters. Here we chose to restrict the cluster size to at most 300 individuals.

The maximum likelihood estimates for the parameters α, β, and σ^2 involve inverting the kinship matrix. If there are n individuals in the cluster, then this inversion requires $O(n^3)$ calculations, but since these calculations only need to be performed once the computational cost of doing a GWAS will only be $O(n^2)$ calculations; the cost of calculating the maximum likelihood estimates if the kinship matrix has already been inverted.

Heritability estimation

The heritability of B_{12} and folate levels was estimated as twice the correlation between sibling pairs. The standardized residual measurements described above were used for the estimation of correlation.

Fraction of variance explained

The fraction of variance explained was calculated using the formula 2f (1 – f) a^2, where f is the frequency of the variant and a is its additive effect. For the calculation of the fraction of variance explained we used the reported estimates from Table 1, 2 and 3. For the secondary variants (Table 3), the adjusted effect was used in the calculation. For B_{12}, the fraction of variance explained is estimated to be 6.3%. For folate, the corresponding value is estimated to be 1.0%.

Effective sample size estimation

In order to estimate the effective sample size of the quantitative trait association analyses, we compared the variances of the logistic and generalized linear regression parameter estimates based on the genealogy imputed genotypes to their one step imputation counterparts. For the quantitative trait association

analysis, assume that a single step imputation (SNVs are imputed, but genealogically imputed genotypes are not used) association analysis with n_1 subjects leads on average to an estimate of the regression parameter with variance σ_1^2 and that the corresponding genealogically imputed genotype association analysis leads to an estimate of the regression parameter with variance σ_2^2, then assuming that variance goes down linearly with sample size we estimate the effective sample size in the genealogically imputed genotype association analysis as $n_2 = \frac{\sigma_1^2}{\sigma_2^2} n_1$. We estimated the effective sample sizes for the Icelandic data to be 23,493 individuals with serum B_{12} measurement and 20,542 with serum folate measurement taking the genomic control (GC) in to account (B_{12} GC: 1.21, folate GC: 1.11) (Supplementary Table 1).

Secondary traits in deCODE Genetics database.

The deCODE Genetics phenotype database comprises medical information on a variety of diseases and traits obtained through collaboration with specialists in each field. This includes information on cardiovascular diseases (myocardial infarction, coronary arterial disease, peripheral arterial disease, atrial fibrillation, sick sinus syndrome and stroke), metabolic disorders (obesity, type 2 diabetes, and metabolic syndrome), psychiatric disorders (schizophrenia, bipolar disorder, anxiety and depression), addictions (nicotine, alcohol), inflammatory diseases (rheumatoid arthritis, lupus, and asthma), musculoskeletal disorders (osteoarthritis, osteoporosis), eye diseases (glaucoma), kidney diseases (kidney stones, kidney failure) and many types of cancers (29 types). Anthropometric measures have also been collected through several of these projects. Routinely measured traits during patients work up (sodium, potassium, bicarbonate, calcium, phosphate, creatinine, blood cell counts, hemoglobin, hematocrit, iron, vitamins, lipids and more) were obtained from the Landspitali University Hospital, Reykjavik, and the Icelandic Medical Center (Laeknasetrid), Reykjavik, between the years 1990 and 2010, in addition to more specific hormonal measures (adrenal hormones, thyroid hormones, and sex hormones). The measurements were normalized to a standard normal distribution using quantile normalization and then adjusted for sex, year of birth and age at measurement. For individuals for which more than one measurement was available we used the average of the normalized value.

The number of independent and uncorrelated secondary traits tested amounts to about 400. Given that we tested 18 markers applying both the multiplicative and the recessive model the threshold of significance is about 3.5×10^{-6}.

REFRENCES

1. Li H, Durbin R (2009) Fast and accurate short read alignment with Burrows-Wheeler transform. Bioinformatics 25: 1754-1760.

2. Li H, Handsaker B, Wysoker A, Fennell T, Ruan J, et al. (2009) The Sequence Alignment/Map format and SAMtools. Bioinformatics 25: 2078-2079.

3. Kong A, Masson G, Frigge ML, Gylfason A, Zusmanovich P, et al. (2008) Detection of sharing by descent, long-range phasing and haplotype imputation. Nat Genet 40: 1068-1075.

4. Kong A, Thorleifsson G, Gudbjartsson DF, Masson G, Sigurdsson A, et al. (2010) Fine-scale recombination rate differences between sexes, populations and individuals. Nature 467: 1099-1103.

5. Marchini J, Howie B, Myers S, McVean G, Donnelly P (2007) A new multipoint method for genome-wide association studies by imputation of genotypes. Nat Genet 39: 906-913.

6. Lander ES, Green P (1987) Construction of multilocus genetic linkage maps in humans. Proc Natl Acad Sci USA 84: 2363-2367.

7. Kruglyak L, Lander ES (1998) Faster multipoint linkage analysis using Fourier transforms. J Comput Biol 5: 1-7.
